# Varicella-zoster virus early infection but not complete replication is required for the induction of chronic hypersensitivity in rat models of postherpetic neuralgia

**DOI:** 10.1371/journal.ppat.1009689

**Published:** 2021-07-06

**Authors:** Benjamin E. Warner, Michael B. Yee, Mingdi Zhang, Rebecca S. Hornung, Benedikt B. Kaufer, Robert J. Visalli, Phillip R. Kramer, William F. Goins, Paul R. Kinchington

**Affiliations:** 1 Department of Ophthalmology, University of Pittsburgh, Pittsburgh, Pennsylvania, United States of America; 2 Department of Microbiology and Molecular Genetics, University of Pittsburgh, Pittsburgh, Pennsylvania, United States of America; 3 Department of Biomedical Sciences, Texas A&M University College of Dentistry, Dallas, Texas, United States of America; 4 Department of Veterinary Medicine, Freie Universität Berlin, Berlin, Germany; 5 Department of Biomedical Sciences, Mercer University School of Medicine, Savannah, Georgia, United States of America; Stanford University Medical School, UNITED STATES

## Abstract

Herpes zoster, the result of varicella-zoster virus (VZV) reactivation, is frequently complicated by difficult-to-treat chronic pain states termed postherpetic neuralgia (PHN). While there are no animal models of VZV-induced pain following viral reactivation, subcutaneous VZV inoculation of the rat causes long-term nocifensive behaviors indicative of mechanical and thermal hypersensitivity. Previous studies using UV-inactivated VZV in the rat model suggest viral gene expression is required for the development of pain behaviors. However, it remains unclear if complete infection processes are needed for VZV to induce hypersensitivity in this host. To further assess how gene expression and replication contribute, we developed and characterized three replication-conditional VZV using a protein degron system to achieve drug-dependent stability of essential viral proteins. Each virus was then assessed for induction of hypersensitivity in rats under replication permissive and nonpermissive conditions. VZV with a degron fused to ORF9p, a late structural protein that is required for virion assembly, induced nocifensive behaviors under both replication permissive and nonpermissive conditions, indicating that complete VZV replication is dispensable for the induction of hypersensitivity. This conclusion was confirmed by showing that a genetic deletion recombinant VZV lacking DNA packaging protein ORF54p still induced prolonged hypersensitivities in the rat. In contrast, VZV with a degron fused to the essential IE4 or IE63 proteins, which are involved in early gene regulation of expression, induced nocifensive behaviors only under replication permissive conditions, indicating importance of early gene expression events for induction of hypersensitivity. These data establish that while early viral gene expression is required for the development of nocifensive behaviors in the rat, complete replication is dispensable. We postulate this model reflects events leading to clinical PHN, in which a population of ganglionic neurons become abortively infected with VZV during reactivation and survive, but host signaling becomes altered in order to transmit ongoing pain.

## Author summary

Acute and chronic pain are common complications of herpes zoster, but the mechanisms underlying the transition to chronic pain states remain poorly understood. Varicella-zoster virus (VZV)-inoculated rats develop persistent behaviors that indicate the development of prolonged hypersensitivity that may model postherpetic neuralgia (PHN) seen in the clinic. To address the requirements of viral gene expression and replication for the induction of pain, we developed mutant VZV and applied them to rat models of PHN. Our results reveal the production of essential VZV regulatory proteins is required to induce nocifensive behaviors, but that full productive virus replication is dispensable. These data suggest a mechanism for pain induction that involves the early expression of VZV regulatory proteins in abortively infected neurons after herpes zoster that may cause aberrant host pain signaling and PHN.

## Introduction

The human neurotropic herpesvirus, varicella-zoster virus (VZV), causes varicella (chickenpox) upon primary infection and herpes zoster (shingles, HZ) following reactivation from a latent state that was established during primary infection [1]. HZ will occur in about one-third of unvaccinated individuals in their lifetime and remains a major public health concern, owing to an expanding aged demographic that are at risk of HZ disease and morbidities. Incidence and disease severity increase significantly with advancing age and/or immune impairment [2]. While incidence of both primary and reactivated disease can be greatly reduced by vaccination [3–6], most adults remain at risk for HZ because they harbor latent, wild-type VZV within sensory ganglia, and uptake of HZ vaccines in the US is far from optimal [7]. In many countries, use of HZ vaccines is minimal or non-existent [8,9], despite providing reasonable rates of protection from HZ and its debilitating consequences. Therefore, research continues into improved treatment and prevention strategies for HZ.

Pain is the most common complication of HZ and is one of the largest contributors to its morbidity. Pain may occur before rash development (prodrome), but more than 80% of individuals over 60-years with HZ will experience pain during and/or after the rash that requires prescription medication treatment [10]. A significant fraction of HZ patients will progress to debilitating chronic pain states known as postherpetic neuralgia (PHN), defined by many as pain lasting more than three months after the typical dermatomal rash of HZ has resolved [11]. Symptoms of PHN commonly include allodynia, defined as hypersensitivity to normally innocuous stimuli that frequently persists after stimulus removal. PHN may also include increased sensitivity to thermal stimuli (hyperalgesia) [12]. PHN incidence rates have been reported to be as high as 20–30% of those with HZ, with greater severity linked to certain dermatomes, rising age, and decline of immune status [8]. Because clinical PHN lasts well beyond visible signs of skin disease, it may not reflect continuous viral replication but rather tissue damage sustained during active HZ disease that propagates ongoing pain signaling. Clinical observations suggest VZV triggers numerous and long-lasting changes indicative of neuropathic damage within innervating sensory tissues [13]. Further, the severity of PHN directly correlates with preceding HZ symptom severity [11], suggesting a “more damage, more pain” scenario. A key difference between acute and chronic pain is response to antiviral treatment. The antiviral acyclovir can often effectively limit acute HZ and associated pain when delivered in a timely manner [14], and treatment during acute HZ may also reduce the duration of subsequent PHN [15]. However, administration of antivirals during PHN is ineffective in most cases [16], reinforcing the concept that chronic pain signaling is not a consequence of ongoing VZV replication. The processes involved are not entirely clear and the mechanisms governing the transition from acute to chronic pain states remain ill-defined. Notably, HZ involves extensive intra-ganglionic spread to result in the infection of large numbers of neurons and non-neuronal cells within the reactivating ganglion [17]. These mediate virus delivery to the periphery by many neurons, and also cause inflammation both at the ganglia and at the skin that contribute to pain [18,19]. Postmortem studies of cadaver ganglia with ongoing HZ at time of death reveal extensive ganglionitis and tissue damage [20,21]. Many complex mechanisms underlying PHN pain have been proposed but few have been experimentally substantiated [12,21,22].

The lack of a reliable small animal model of VZV reactivation and HZ disease has precluded the study of VZV reactivation-driven pain. Rodent models show little to no clinical presentation after infection. Even non-human primate models using the closely related simian varicella virus (SVV) are difficult to employ for HZ and pain studies due to inefficient virus reactivation. Even then, animals do not routinely develop signs of PHN. Several groups have used the rat to investigate VZV latent states [23–25]. It was then demonstrated by multiple groups that Wistar and Sprague-Dawley rat strains inoculated with VZV at the footpad develop prolonged signs of pain that could serve as preclinical models for exploring mechanisms and treatment strategies for PHN [22]. The models involve subcutaneous inoculation of cell-associated VZV into the rat hind footpad [26–32] or more recently, the whisker pad [33–38]. While animals show no outward signs of skin infection, inflammatory response, or disease, they develop nocifensive behaviors lasting several weeks. It has never been thoroughly resolved if VZV productive replication occurs within inoculated rats and if this is a requirement for nocifensive responses. Work from our group indicated that VZV did not replicate in rat primary cell cultures, suggesting VZV replication *in vivo* is unlikely [32]. One *in vivo* study indicated that VZV-induced hypersensitivity in rats was unresponsive to acyclovir administration [27]. Our group reported that rats inoculated with UV-inactivated VZV did not develop long-term nocifensive behaviors, suggesting a requirement for viral gene expression in the development of pain behaviors [31]. VZV has been shown to induce subtle changes in host gene expression within infected ganglia [32]. Ganglionic sections of rats with hypersensitivity show sporadic staining for the major VZV transcriptional regulatory protein, IE62 [28–30,34]. Taken together, these results suggest that VZV may initiate abortive infections in the rat that nevertheless induce nocifensive behaviors and hypersensitivity.

**Fig 1:**
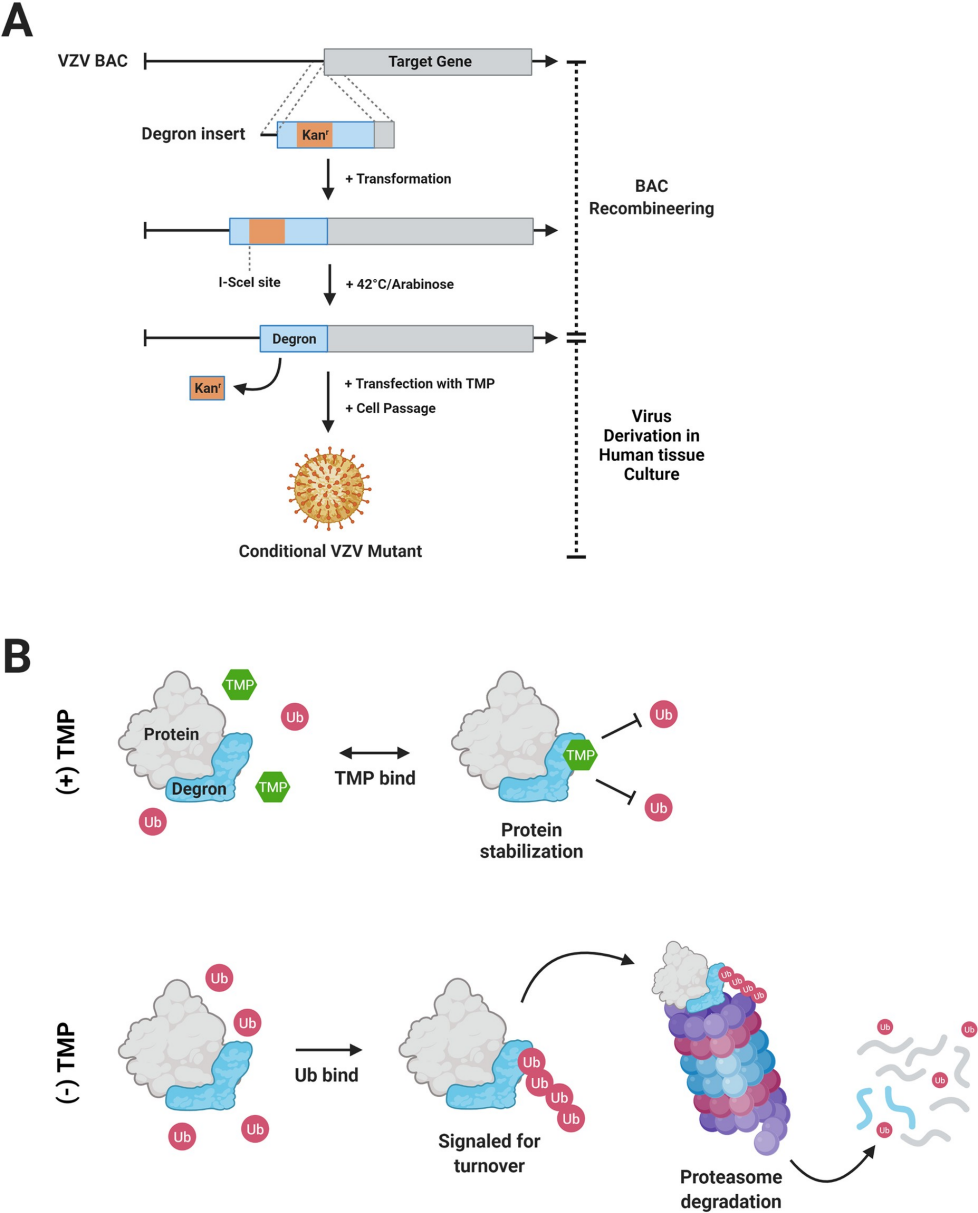
Overview of VZV BAC development with a degron insertion and TMP-dependent protein turnover. (A) A target gene in the VZV BAC is engineered by recombining a degron sequence with an interrupting kanamycin resistance cassette (kan^r^) that allows for positive selection in *E*. *coli* GS1783 as detailed in the methods. A second induced recombination event in conjunction with expression of the homing endonuclease I-*Sce*I results in markerless excision of the kan^r^ cassette, so that the degron coding protein is fused to the target gene ORF. BACs are then transfected into human TRPE cell monolayers in the presence of the stabilizing ligand trimethoprim (TMP) to yield infectious VZV. (B) In the presence of TMP (top), TMP (green) is thought to bind the degron and prevent ubiquitin (Ub, red) ligation, thus stabilizing protein and halting turnover. In the absence of TMP (bottom), Ub is ligated to the degron, and the entire protein is targeted for degradation by ubiquitin-proteasome pathway. Created with BioRender.com.

Here, we further addressed requirements of VZV replication and gene expression in development of pain indicators in the rat PHN models. We developed and used novel VZV mutants that show conditional replication, depending on the stability of specific essential proteins containing a “degron” domain fused to either the amino- or carboxy-termini of the target protein (Fig 1) [39]. Turnover of degron-protein fusions is regulated by a cell permeable drug, trimethoprim (TMP), that stabilizes the degron domain and prevents proteasomal degradation. We targeted VZV proteins that have essential roles at the beginning and end of the lytic herpesvirus gene expression cascade. All herpesviruses express their genes in an ordered manner in which immediate-early (IE or α) genes are expressed first, followed by early (E or β) genes that encode proteins that generally act to replicate viral DNA, and then late (L or γ) genes whose proteins are generally involved in virus assembly and egress [40]. VZV IE gene encoded proteins have regulatory functions that subsequently control the rest of the viral gene expression program [40–44]. Using a degron domain from the *E*. *coli* dihydrofolate reductase (DHFR), we developed three conditionally replicating VZV recombinants. These were used in conjunction with a cell-complemented VZV deletion mutant that did not express the essential ORF54 gene encoding the capsid portal protein [45]. The VZV recombinants were then assessed for their ability to induce behavioral hypersensitivities in rats when inoculated in the presence or absence of TMP. We show that VZV blocked at late stages of assembly and full productive replication in the rat still induced prolonged hypersensitive behaviors, establishing that productive replication is not required. In contrast, rats inoculated with conditionally replicating VZV with a degron attached to IE regulated proteins only developed hypersensitivity when the inoculates were supplemented with TMP to stabilize the targeted degron proteins. This suggested that the expression of essential IE transcriptional regulatory proteins IE4 and IE63 was required for the stimulation of persistent pain behaviors in the rat. These data are consistent with a hypothesized mechanism in which a limited VZV gene expression program in the rat results in altered host neuronal pain signaling. We discuss that this may occur in patients with PHN, in which an abortive VZV infection process occurs during HZ within sensory neurons that survive reactivation but go on to signal pain.

## Materials and methods

### Ethics statement

All animal studies were performed in accordance with protocols approved by the University of Pittsburgh Institutional Animal Care and Use Committee (IACUC, protocol #18022168). This protocol meets the standards for humane animal care and use as set by the Animal Welfare Act and the NIH Guide for the Care and Use of Laboratory Animals.

### Cells and viruses

VZV parental Oka (pOka) is a wild-type varicella isolate from which the current live attenuated vaccines were derived [46]. It has been established that pOka can induce both mechanical and thermal hypersensitivity in rat models of PHN [31–38] and was used as a positive control in all studies. Recombinant viruses were derived from a pOka bacterial-artificial chromosome (BAC) which contains a self-excisable BAC replicon [47] that was subsequently corrected for two spurious mutations in the VZV ORF40 and ORF50 genes and contained an N terminal GFP reporter fused to ORF23 [48].

Human telomerase (Tert) immortalized RPE-1 (TRPE, ATCC CRL4000) cells were grown in Dulbecco’s Minimal Essential Media (DMEM, Gibco 10569–010) supplemented with 10% fetal bovine serum (FBS, R&D Systems S11150) and an antibiotic/antimycotic mixture (Caisson ABL02). TRPE cells were used for reconstitution of virus from BAC DNAs, VZV propagation, and stock preparation. VZV stocks were made as previously detailed [31]. Briefly, TRPE monolayers grown to 80–90% confluency at 37°C were infected, incubated 48–72 hours at 34°C, and underwent trypsinization and re-plating when needed, until >60% of cells showed visible cytopathic effect, or 95%+ fluorescence positivity where relevant. Virus infected cell stocks were generated by trypsin digestion, concentrated by low-speed centrifugation, then resuspended in cell freeze media (DMEM, 20% FBS, 10% DMSO) and subjected to slow freeze at -80°C, followed by liquid nitrogen storage. Conditional VZV mutants were derived and grown in TRPE cells with the media additionally supplemented with 100 nM TMP (Sigma T7883) [39]. Prior to storage, all conditional VZV samples were washed extensively in cold DMEM without TMP to remove residual drug prior to freezing. Aliquots from liquid nitrogen storage were titrated to assess VZV cell-associated infectious center formation (taken as titer) by dilution and plaque assay in permissive conditions on ARPE-19 cells (ARPE, ATCC CRL2302) using supplemented DMEM as noted above, and TMP when necessary.

### Generation of recombinant VZV

Recombinant VZV ORF54Δ and its complementing ARPE19-ORF54 (A54) cells were detailed previously [45]. Recombinants VZV ORF4nDHFR, VZV ORF63cDHFR, and ORF9cDHFR were constructed by two-step Red-mediated scarless recombination methods as previously described [49], utilizing the VZV pOka BAC that contains green fluorescent protein (GFP) fused to the N-terminus of the minor capsid protein encoded by ORF23 (HSV VP26 homolog), as described previously [48]. Mutagenesis was performed in the GS1783 *E*. *coli* strain (gift of Dr. Gregory Smith, Northwestern University, IL) containing the heat shock-inducible (42°C) λRed recombination system and an L-arabinose inducible I-*Sce*I restriction enzyme. A transfer plasmid for the 480-bp degron domain derived from the *E*. *coli* DHFR gene [39] was generated by the insertion of the I-*Sce*I kanamycin resistance (kan^r^) cassette flanked by 40-bp homologous sequences used for the scarless removal of the selective marker (Fig 1A). PCR primers (Table 1) were designed to anneal at the end of the degron domain sequence and extend the cassette by 40-bp sequences homologous to the targeted insertion sequence in the VZV BAC. The fragment was amplified using the proofreading polymerase PrimeSTAR GXL (Takara Biochemicals, R050A). For generation of VZV ORF63cDHFR, the coding sequence of ORF70 (duplicated ORF63 gene) was replaced by a PCR amplified ampicillin resistance cassette before kan^r^ removal from ORF63cDHFR, and co-selected by growth on plates supplemented with chloramphenicol (to maintain the BAC) kanamycin and ampicillin. All BACs were subjected to extensive characterization by digestion with multiple restriction enzymes to ensure no deletions of BAC DNA sequences, and all in frame gene-degron fusions were verified by Sanger sequencing of PCR amplified fragments across the respective junctions. Infectious virus was reconstituted by the transfection of the recombinant BACs using Lipofectamine 3000 (ThermoFisher) on TRPE cells grown in the presence of 100 nM TMP. VZV were passaged 3–5 times in permissive growth media (supplemented DMEM + 100 nM TMP) to allow self-excision of the BAC sequence, and master stocks were prepared and titrated as previously described. All mutant VZV used in these studies were grown from low passage master stocks with minimum of 8–10 additional passages. Integrity of the inserted DHFR sequences were confirmed by Sanger sequencing across the 5’ and 3’ insert junctions of both the BACs and of the resultant viruses.

**Table 1:** Primer design for VZV BAC recombineering.

#	Gene	Direction	Primer Sequence (5’ ➔ 3’)
1	ORF4	Fwd	AGGCAACTGCAAACACGCAATTGTCAGATATTTTGCAGCCggatccgccaccatgatcagtctgattgcggcgttagcg
		Rev	TCACAAATAGTAGACACGTCTGGGTCGGTTGGAATTGAAGCAGAGGCGCAtgctcgccgctccagaatct
2	ORF9	Fwd	CGTGTTTGGATATTTCACGACCCTATCGTTTATTTACGTAggatccgccaccatgatcagtctgattgcggcgttagcg
		Rev	CATTAGAGCGACAAAGTCTGTCACCGTCGGAAGATGCCATtgctcgccgctccagaatct
3	ORF63	Fwd	AGCCCCGCGCCGGCATGATATACCGCCCCCCCATGGCGTGatgatcagtctgattgcggc
		Rev	AAGACACGAGCCAAACCATTGTATTTATTTATAAAGActatgctcgccgctccagaatct
4	ORF70	Fwd	GTTTTGTTGTGCAGGGTTCGTCCGATTCATAACGCGACAGaaatgtgcgcggaacccctatttg
		Rev	TATCCACAACACCCCACTCCCCCACAGACAGACATCAAAActtggtctgacagttaccaatgctt

Uppercase letters in all rows denote bases that share sequence homology with VZV for directional recombination. Lowercase letters are sequences homologous to the *E*. *coli* DHFR degron insert sequence (#1, 2, 3) or ampicillin resistance cassette sequence (#4).

DNA of recombinant viruses generated from the BACs was Southern blot analyzed by extraction of nucleocapsid VZV DNA from 5 x 175 cm^2^ infected cell flasks showing >70% cytopathic effect using the procedure detailed previously [50]. 1ug of DNA from each VZV recombinant DHFR virus and VZV from the VZV pOka BAC was assessed by restriction digestion with multiple enzymes including *Kpn*I (S1 Fig) and *Sph*I (S2 Fig), subjected to agarose gel electrophoresis, transferred to a nylon membrane (Millipore INYC00010), and then probed to identify the fragments that contain the degron element. The hybridization probe was generated by PCR amplification with oligos homologous to the DHFR degron using the primers 5’-CCTGGTTTAAACGCAACACC and 5’-GTGAGAGTTCTGCGCATCAG to amplify a 474-bp product, which was then labeled with a Biotin DecaLabel kit (ThermoFisher K0651). Probe hybridization (10 ng/mL) was completed overnight at 42°C and detected by binding fluorescent IRDye 800CW Streptavidin (LI-COR 926–32230) for 1-hr at room temperature at a 1:10,000 dilution. The blot was then imaged on a LI-COR Odyssey IR in linear range.

### Virus growth analysis

Growth of VZV recombinants modified with degrons were completed using an infectious focus assay as previously detailed [51] with modifications. Briefly, sub-confluent TRPE monolayers were plated in 12-wells (~3.5x10^5^ cells/well) and infected with cell-associated, pre-titrated VZV at approximately 100 plaque forming units (PFU) per well, in duplicate. Infections were carried out in media containing 100 nM TMP (permissive) or no TMP (nonpermissive) conditions that was then maintained throughout the time of incubation. At times indicated, infected cells monolayers were harvested by trypsinization of the infected cultures and then replating dilutions of the cell suspensions in duplicate on ARPE cells under permissive conditions to quantify the number of infectious cells able to initiate formation of a plaque. Wild-type VZV pOka served as positive control. Titrated infections were fixed at 5-dpi, stained with crystal violet, and plaques were counted on a dissection microscope. Biological sample duplicates and technical duplicates were averaged and graphed.

### Immunoblotting

Immunoblotting methods were previously described [52] and performed with minor modifications. Briefly, sub-confluent TRPE monolayers in 6-well plates (~1 x 10^6^ cells/well) were infected with 1x10^4^ PFU VZV-TRPE cell-associated virus (1:100 infected to uninfected cells) and incubated in DMEM with or without 100 nM TMP for 24-, 48-, and 72-hours post-infection. Cells were harvested by washing twice in ice-cold 1x PBS and removed by mechanical dislocation into 1x PBS containing protease (ThermoFisher Halt) and phosphatase (Roche PhosSTOP) inhibitors. After concentration by centrifugation at 12,000*xg* at 4°C for 5 minutes, cells were resuspended in 100 μl 1x PBS with 2x protease inhibitors, to which 100 μl of 2X SDS PAGE lysis buffer was added for a final sample volume of 200 ul. Samples were briefly probe-sonicated, heated to 95°C for 5 min, then loaded onto precast 4–15% acrylamide gradient SDS-PAGE gels (Bio-Rad Criterion) and run at 65V until completion. Proteins were transferred by electrophoresis to a polyvinylidene difluoride membrane (Millipore Immobolin-FL 00010) overnight at 15V, and membranes blocked overnight at 4°C using LI-COR “Intercept” Blocking Buffer. Blots were incubated with dilutions of primary antibodies at 4°C for 4–24 h in diluted blocking buffer solution containing 0.1% Tween-20, washed extensively in the same buffer, and further incubated with secondary species-specific antibodies linked to near IR dyes (LI-COR, IRDye 680/800) at 1:20,000 dilution for 1-h at room temperature, washed, and imaged on a LI-COR Odyssey IR in a linear range.

### Fluorescent microscopy

For cell localization studies, TRPE monolayers prepared on 4-well chambered slides (Sigma-Aldrich Nunc Lab-Tek II C6807) were infected with 50 or 10 PFU VZV per chamber and incubated 4-days under TMP permissive and nonpermissive conditions at 34°C. Monolayers were fixed by incubation for 20-min in 4% paraformaldehyde at room temperature, washed in 1X PBS, and blocked overnight in 10% heat-inactivated goat serum (HIGS) in PBS. Samples were incubated in HIGS-PBS diluted primary antibodies overnight, washed, and incubated with Alexa Fluor-coupled secondary antibodies for 1-h at room temperature. After a final 10-minute incubation with DAPI, washed chambers were separated from the slides and coverslips mounted. Slide imaging was performed on an Olympus IX83 using a 60X (N.A. 1.25) oil objective in a linear range. These images were processed using Olympus CellSens software and ImageJ, and comparative images were processed equally.

Plaque sizes were determined by imaging VZV infectious centers at 4-dpi prepared under permissive and nonpermissive conditions and grown at 34°C. Monolayers were fixed by 20-min incubation in 4% paraformaldehyde at room temperature, washed in PBS, and stored at 4°C in PBS until imaged. All viruses, except for pOka, produce a GFP fused ORF23 protein (ORF23p) that served to identify VZV infectious centers by fluorescent microscopy. To image pOka plaques under similar conditions, infected samples were probed with a primary antibody to ORF23p and detected with Alexa Fluor 488 secondary antibody as described in the localization analysis method. Images containing individual plaques were each acquired under identical acquisition settings with Cell Sens software on an Olympus IX83 microscope with a 10X (N.A..030) air objective. Images were exported from Olympus CellSens software and analyzed in Metamorph (Version 7.7, Molecular Devices, San Jose, CA). Data was reported as area in pixels (1 px = 1.024 μM).

### Antibodies

Primary mouse antibodies to proteins IE4 (HR-VZV-20), ORF9p (HR-VZV-38), ORF23p (HR-VZV-12), and IE63 (HR-VZV-33) were acquired commercially from Center for Proteomics, University of Rijeka (CapRi). gE (SC-56995) was acquired from Santa Cruz Biotechnology (Dallas, TX, USA) and β-actin (A00702) was purchased from GeneScript (Piscataway, NJ, USA). Rabbit antibody to α-Tubulin (600-401-880) was purchased from Rockland Immunochemicals (Limerick, PA, USA). Rabbit antibodies to IE62, ORF29p, and IE4 were reported previously [53–55].

Secondary IRDye antibodies for immunoblotting compatible with the LI-COR Odyssey were purchased from LI-COR: goat anti-mouse 680CW (926–32220), goat anti-mouse 800CW (926–32210), goat anti-rabbit 680CW (926–32221), goat anti-rabbit 800CW (926–32211). Those used for immunohistochemistry were purchased from Invitrogen: Alexa Fluor 488 goat anti-mouse (A11029), Alexa Fluor 546 goat anti-mouse (A11030), Alexa Fluor 488 goat anti-rabbit (A11034), Alexa Fluor 546 goat anti-rabbit (A11035).

### Nucleic acid analyses of animal DRG tissues by RT-qPCR

Messenger RNA transcripts encoding ORF4, ORF62, ORF63, or DHFR sequences were quantified from rat DRG following footpad inoculation and tissue harvest as detailed previously [32], using reverse transcription quantitative real-time polymerase chain reaction (RT-qPCR) and TaqMan probes (S1 Table). Briefly, male Sprague-Dawley rats (n = 24) were divided into four groups for inoculation (pOka, VZV ORF4nDHFR, VZV ORF63cDHFR, and uninoculated) and sub-divided into three timepoint groups for post-infection harvest at 4-, 5-, and 7-dpi. Rats (n = 6/group) received injections of 2x10^5^ PFU VZV into the glabrous footpad. At each timepoint, rats (n = 2/group) were necropsied, L4, L5, L6 DRG were micro-dissected and snap frozen in liquid nitrogen until nucleic acid purification. RNA was purified by mechanically disrupting tissues in TRIzol (ThermoFisher) reagent with a PT1200E tissue homogenizer (Kinematica, Bohemia, NY) for 10-s at ~75% power. RNAs were dissolved in nuclease free H_2_O, DNase-treated (ThermoFisher EN0521) and converted into cDNA using a High-Capacity RNA-to-cDNA kit (Applied Biosystems 4387406). cDNAs were analyzed by thermocycling (95°C, 60°C, 40X) in an Applied Biosystems StepOne Plus qPCR system with PrimeTime Gene Expression Master Mix (IDT 1055770). Gene expression was detected by TaqMan assay and relative expression values were calculated by the 2^-ΔΔCt^ method with comparison to rat GAPDH (Applied Biosystems Rn01775763_g1) and displayed as fold-change over GAPDH (equal to 1). Data are averaged results from two identical qPCR assays. ORF62 and ORF63 TaqMan primer sets (S1 Table) have been described [56].

### Animal studies

Male Sprague-Dawley rats (Charles River Laboratories) weighing 200–250 grams were acclimated to housing and mechanical and thermal behavioral measurement conditions for 1–2 weeks until establishing a consistent behavior baseline that was in accordance with historical records of previous studies. Animals falling outside the established parameters were excluded. All inoculations and behavioral assessments were blinded so the behavioral response recorder was ignorant of the inoculum source and injection location. Cell-associated VZV for animal inoculation was prepared by fast thawing pre-titrated virus aliquots stored in liquid nitrogen, followed by centrifugation at 150*xg* at 4°C for 10 minutes, an ice-cold 1x PBS wash to remove residual freeze media, and resuspension in 1x PBS for a final concentration of 4x10^6^ PFU/ml. Washed, intact cell-associated virus was maintained on ice for no more than 1-h prior to foot or whisker pad inoculation. Uninfected cell equivalents were prepared identically. When TMP was included in the inoculum, virus infected cells were resuspended at the appropriate concentration in ice-cold 1x PBS supplemented to 500 nM TMP.

In the footpad model, inoculations were carried out as detailed previously [31] with slight modifications. Briefly, gently restrained rats were subcutaneously inoculated with 25–50 μl containing 2x10^5^ PFU VZV into the glabrous skin of the left or right rear footpad. Whisker pad inoculations were as detailed previously [34] using isoflurane anesthetized rats whereupon virus was injected subcutaneously in the right or left whisker pad. Animals were monitored for recovery and returned to housing. Mechanical and thermal responses of the footpad were performed as detailed previously [31]. Briefly, mechanical allodynia (MA) at the footpad was assessed using a set of calibrated von Frey monofilaments (Stoelting Company, Wood Dale, IL). Animals were focus-distracted by presenting fruit cereal throughout the procedure [57]. Response measurement began with the 10-gram (evaluator size 5.07) filament and measured six times each on the injected (ipsilateral) and non-injected (contralateral) glabrous rear footpads through a metal grid stage. The monofilaments were applied using the “Up-Down” method [58]. A maximum von Frey filament weight of 180-grams was employed. Data was calculated as 50%-gram weight threshold and presented as withdrawal threshold average in grams. Thermal hyperalgesia (TH) measurements utilized a Hargrave’s apparatus (IITC, Woodland Hills, CA) and followed the Hargrave’s method after a minimum 24-h rest period following MA measurements [59]. Briefly, rats were placed on a stable 32°C glass stage in a partitioned acrylic box and allowed to acclimate for 10-minutes prior to measurement. A concentrated light source at consistent distance from under the glass stage was applied to the center area of each rear footpad, and the time to withdrawal for each rear footpad was recorded in seconds, with a maximum cut-off time of 30-seconds to avoid tissue damage. Each footpad was assessed four times in sequential order, testing only the ipsilateral or contralateral footpad per pass, allowing several minutes between measurements of a single footpad to avoid residual sensitivity issues. Data is presented as the average withdrawal time per footpad in seconds.

For assessment of facial affective pain responses, the Fuchs place-escape-avoidance-paradigm (PEAP) method was used as previously described [34,60]. Briefly, rats were placed in a 30 x 30 x 30-centimeter clear, acrylic box in which half was made opaque by attaching a black cloth to the exterior. Rats were allowed to acclimate to box for 10-minutes, during which rats unanimously chose to remain on the dark side. A 60-gram (evaluator size 5.88) von Frey monofilament was applied to the left or right whisker pad, depending on the rat’s location in the box, every 15-seconds for a total of 30-minutes. Monofilament was applied to the ipsilateral (inoculated) whisker pad if the rat’s head was on the dark side of the enclosure and applied to the contralateral whisker pad if rat’s head was on the clear side. Uninoculated rats chose to remain on the dark side, while inoculated rats showed reduced time on the dark side if the von Frey monofilament stimulation was noxious. A positive nocifensive response is defined by preference for the clear side of the box after repeated stimulation of the ipsilateral whisker pad on the dark side. The measurements are presented as time spent on the dark side in 5-minute binned increments over a 30-minute period.

### Statistics

**Fig 2:**
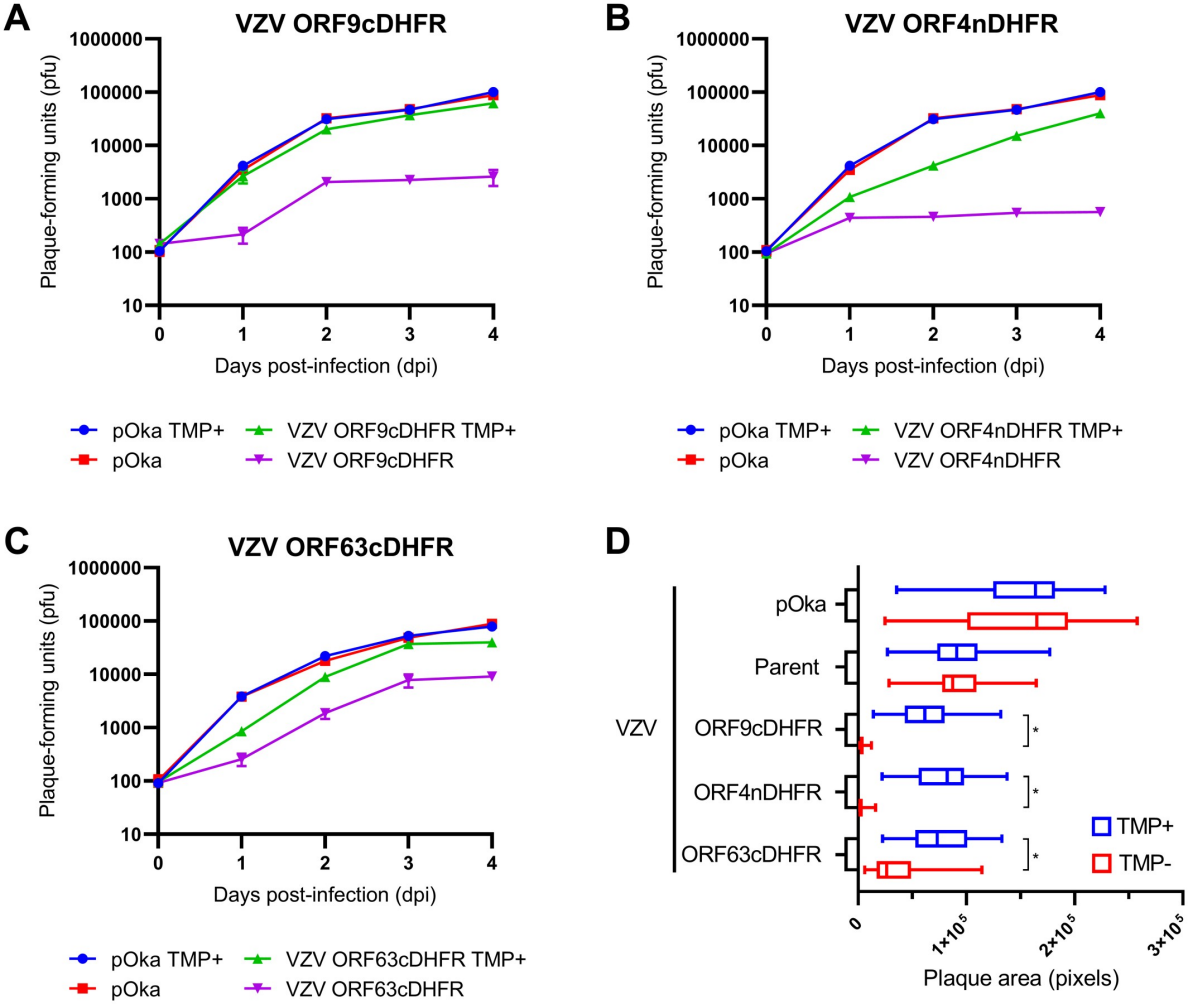
Growth of VZV containing DHFR degron sequence on specific genes confers TMP-conditional virus growth and formation of plaques. (A-C) Conditional growth analysis of three VZV after initiating the infection of monolayers at low multiplicity with VZV degron virus or pOka. Cultures were grown in the presence or absence of 100 nM TMP maintained throughout the growth period from 0–4 days post-infection (dpi). Each day, cultures were then trypsinized and the infectious cell titer was determined by growth on new monolayers in conditions in which TMP was provided in the media. Shown are growth analyses for (A) VZV ORF9cDHFR, (B) VZV ORF4nDHFR, and (C) VZV ORF63cDHFR. (D) Plaque size of wild-type VZV (pOka) or VZV derived from the parental BAC (parent) or containing the degron addition to the respective genes compared under permissive (100 nM TMP, blue) or nonpermissive (0 nM TMP, red) conditions at 4-dpi. Images (n = 28–35) were acquired for each virus/condition under identical settings and evaluated as detailed in methods and presented in pixel size estimated from images acquired under 10X (NA.030) air objective. Error bars: SD.

All statistical tests were performed on Prism 9 software (GraphPad, La Jolla, CA). Error bars are represented as standard deviation (SD), or standard error of the mean (SEM) as noted in the figure legends. Unpaired T-tests with Welch’s correction were performed on the plaque size data (Fig 2D). Two-way ANOVAs were performed for each behavioral assessment (Figs 7–11) with a Bonferroni post-hoc correction.

## Results

### Development and characterization of conditionally replicating VZV with degron fusion to essential VZV proteins

The hypothesis that a VZV limited gene expression profile was required for the development of nocifensive behaviors in the rat models of PHN was only partly answered by our previous studies [32]. In that work, VZV was shown to infect primary rat cell cultures and undergo some virus gene expression, but VZV did not spread beyond the first round. We reasoned that such cultures may not represent the many cell types that could be potentially infected and support VZV *in vivo* in rats and showing a lack of VZV growth in all rat tissues *in vivo* is a difficult task. We considered that a more definitive approach to test our hypothesis would be to evaluate rats inoculated with VZV mutants that are genetically unable to proceed past certain stages of the virus replication cycle. While recombinant VZV with essential gene mutations have been described [47,61,62], their growth requires complementation and for reasons unclear, genetically stable VZV-permissive lines harboring VZV genes have proved to be more difficult to generate than those used for the development of HSV-1 mutants. Complementation of previously detailed VZV deleted for ORF9 [47] and ORF4 [63] was achieved by infection with high titer baculovirus containing a CMV-IE promoter-driven VZV gene, with concurrent treatment with sodium butyrate to inhibit type I histone deacetylases. This approach that was not amenable to apply to the rat models of PHN and did not yield the high titers required in the models. Therefore, we adopted an alternative approach in which replication-conditional VZV were made using a protein degron fusion system [39]. The 480-bp coding degron motif used was derived from *E*. *coli* DHFR and was added by recombination to fuse to several target VZV genes in frame within the virus. The DHFR degron facilitates protein turnover unless it is stabilized by the cell-permeable ligand TMP (Fig 1A). In the absence of TMP, the protein is turned over as a result of ubiquitin binding to the degron, which initiates proteasome-mediated turnover of the fused protein (Fig 1B). The system overcame the need to generate complementing cells and was successfully exploited to show that ORF63 protein is essential for SVV growth [64]. Unexpectedly, VZV with the degron fused to ORF62 (with concurrent deletion of the reiterated ORF71) encoding the major transcriptional regulator protein IE62 were found to be not TMP-growth conditional. Several additional VZV BACs with the degron fused to candidate VZV proteins involved in DNA replication (some evaluated as both amino and carboxyl fusions) did not yield functional virus from 3 separate transfections of 4–6 independently developed BAC constructs per virus (Table 2). This suggested that the degron addition was not compatible with some essential protein functions and that not all VZV genes can be analyzed by the degron approach.

**Table 2:** Developed VZV BAC and degron containing VZV recombinants.

Gene	Protein	Function	Terminus attached	Resulting VZV
ORF4	IE4	regulator of mRNA export	N	replication conditional
ORF6	ORF6p	DNA primase	C	not viable
ORF9	ORF9p	tegument protein	C	replication conditional
ORF10	ORF10p	transcriptional activator	N or C	not replication conditional
ORF29	ORF29p	ssDNA binding protein	N or C	not viable
ORF52	ORF52p	DNA helicase	C	not viable
ORF55	ORF55p	DNA helicase	C	not replication conditional
ORF61	ORF61p	transcriptional regulator	N or C	not viable
ORF62	IE62	major transcriptional activator	N or C	not replication conditional
ORF63	IE63	transcriptional regulator	C	replication conditional

Three VZV recombinants were subsequently found to show TMP-dependent growth, one being VZV with the degron inserted at the 3’ end of ORF9. This ORF encodes ORF9p, an essential phosphorylated late-expressed protein (orthologous to HSV-1 VP22) that predominantly localizes to the cytoplasm [65] and interacts with several structural proteins at the *trans*-Golgi network (TGN) during late infection [66]. Evidence suggests that ORF9p has key roles in tegument formation and secondary envelopment [67]. This VZV (VZV ORF9cDHFR) replicated similar to wild-type pOka VZV when grown in media supplemented with 100 nM TMP but showed a near 2-log reduction in the number of infected cell progeny by 48-h when TMP was withheld from the growth media (Fig 2A). Growth of VZV pOka was not influenced by the presence or absence of TMP. A similar result was found for VZV with the degron attached to the 5’ end of ORF4, which has been reported to be expressed as an immediate-early gene [41]. ORF4 is an essential gene and its protein (IE4) regulates VZV gene expression at the post transcriptional levels that involve the nuclear export of viral intronless mRNA [68,69]. IE4 has nuclear and cytoplasmic distribution in infected cells and interacts with SR nuclear shuttling proteins [69]. The virus (VZV ORF4nDHFR) showed a slight reduction of growth in the presence of TMP when compared to wild-type pOka virus (Fig 2B), but when grown in the absence of TMP, highly reduced progeny virus yields were detected at day 1 and longer times. This suggested that the addition of the degron to the protein may have a subtle effect on IE4 function but did not impair its essential functions. The third growth conditional VZV contained the DHFR degron domain inserted at the 3’ end of ORF63, in a similar manner to that done previously with SVV IE63 [64]. ORF63 encodes the protein IE63, which has essential viral and host gene regulatory functions [42]. Since ORF63 lies within the reiterated genome sequences and is duplicated as ORF70, the BAC was deleted for ORF70 by replacing it with an ampicillin resistance cassette (amp^r^). The resulting virus (VZV ORF63cDHFR) showed significantly reduced virus replication and production of virus progeny over a 4-day time course in the absence of TMP, with approximately 2/3^rd^ to 1.2-log difference without TMP compared to virus growth with TMP (Fig 2C).

The three viruses were characterized to verify the expected degron insertion and its stability in the virus. This included DNA sequencing of a PCR amplified fragment spanning the fusion of each virus to confirm the in-frame fusion, and analyses of viral DNA obtained from nucleocapsids obtained from virus grown in the presence of 100 nM TMP in the media. The DNA was subjected to gel analyses and Southern analysis using a DHFR degron-specific probe sequence. This was particularly important to characterize VZV ORF63cDHFR and show that ORF63 had replaced ORF70 by homologous recombination and removed the BAC replicon sequences (S1 Fig). The degron-specific probe hybridized to a large DNA fragment of the *Kpn*I digested VZV genomic DNA for ORF9cDHFR and ORF4nDHFR, but for VZV ORF63cDHFR, two *Kpn*I digested DNA fragments showed a 480-bp increase in size and hybridized the DHFR probe. These bands were of the sizes expected for not only ORF63, but also for its replacement of the ampicillin cassette in the ORF70 BAC used to derive VZV ORF63cDHFR. The blots and fragment sizes also demonstrated the expected recombinational removal of the BAC replicon from the virus, as originally designed [47]. The virus DNAs were likewise assessed by digestion with *Sph*I (S2 Fig), which gave a novel fragment for each virus that confirmed the correct insertion of the degron sequence at the target site.

We next addressed growth by plaque size formation for the three degron viruses in parallel to pOka under identical conditions in the presence or absence of TMP (Fig 2D). Approximately 30 plaques per virus per condition were imaged for each virus/condition and analyzed by integrated morphometry analysis. Plaques formed by pOka and the parental BAC derived VZV (parent) were identical when grown with or without TMP, establishing that TMP does not affect WT VZV growth; parent plaque size trended to be slightly smaller than those formed by wild-type pOka on TRPE cells. VZV plaques formed by the degron-containing viruses showed a similar marginally reduced average plaque size under permissive conditions compared to pOka, but under nonpermissive conditions, the plaques were barely detectable and involved only a few cells in the absence of TMP for VZV ORF9cDHFR and ORF4nDHFR. VZV ORF63cDHFR showed greatly reduced plaque size but appeared to show a slightly leaky growth restricted phenotype as seen in the timed growth curve analyses (Fig 2C).

**Fig 3:**
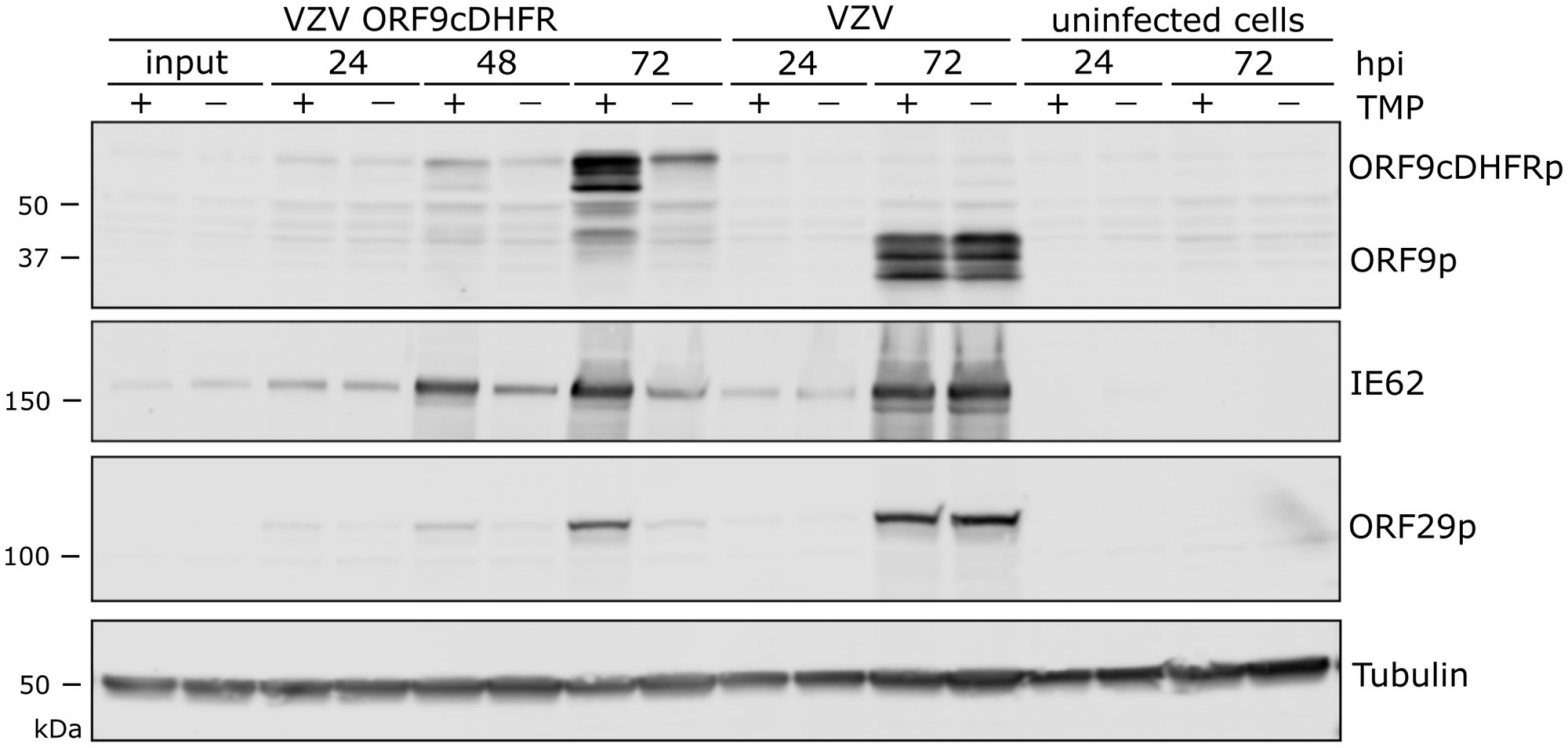
Proteins produced by VZV ORF9cDHFR and pOka grown under media containing or without TMP. (A) Cells were infected with titrated virus infected cell stocks as detailed in methods to allow multi-step virus growth analysis with VZV ORF9cDHFR or pOka. Proteins in infected cultures were harvested at times 24, 48 or 72-hpi in the presence (+) and absence (-) of 100 nM trimethoprim (TMP) and compared to uninfected cell extracts. SDS PAGE separated proteins were immunoblotted and probed with antibodies to ORF9, or proteins from other herpesvirus kinetic classes (IE62, ORF29p), and a cellular control (alpha-tubulin). Signals were determined using a LICOR Odyssey IR imager. ORF9 shows multiple species due to several recognized phosphorylated forms, and the expected size increase (~18 kDa) due to degron motif addition. The sizes of marker proteins (kDa) are indicated to the left of the respective blots. The blots are scanned in linear range and are representative of two identical experiments.

**Fig 4:**
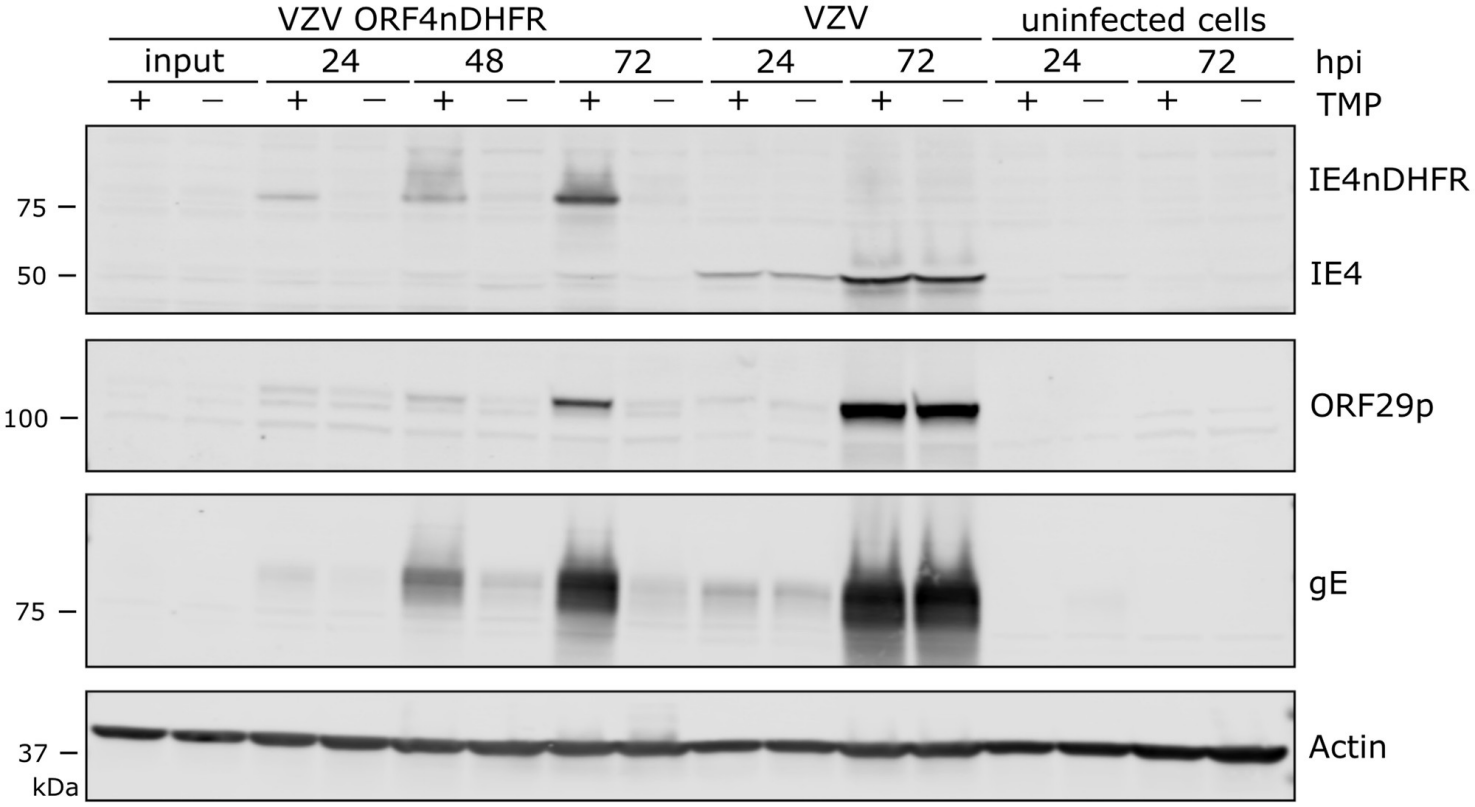
VZV IE4 is regulated by DHFR degron domain fused to amino-terminus. VZV ORF4nDHFR was analyzed in a manner similar to that detailed for VZV ORF9cDHFR in the image and legend for Fig 3, with the exception that additional proteins were identified using antibodies to VZV gE and cellular protein actin. Proteins were from samples grown under TMP permissive (+) and nonpermissive (-) conditions and compared to wild-type pOka infections and uninfected cell equivalents. The blots are scanned in linear range and are representative of two identical experiments.

**Fig 5:**
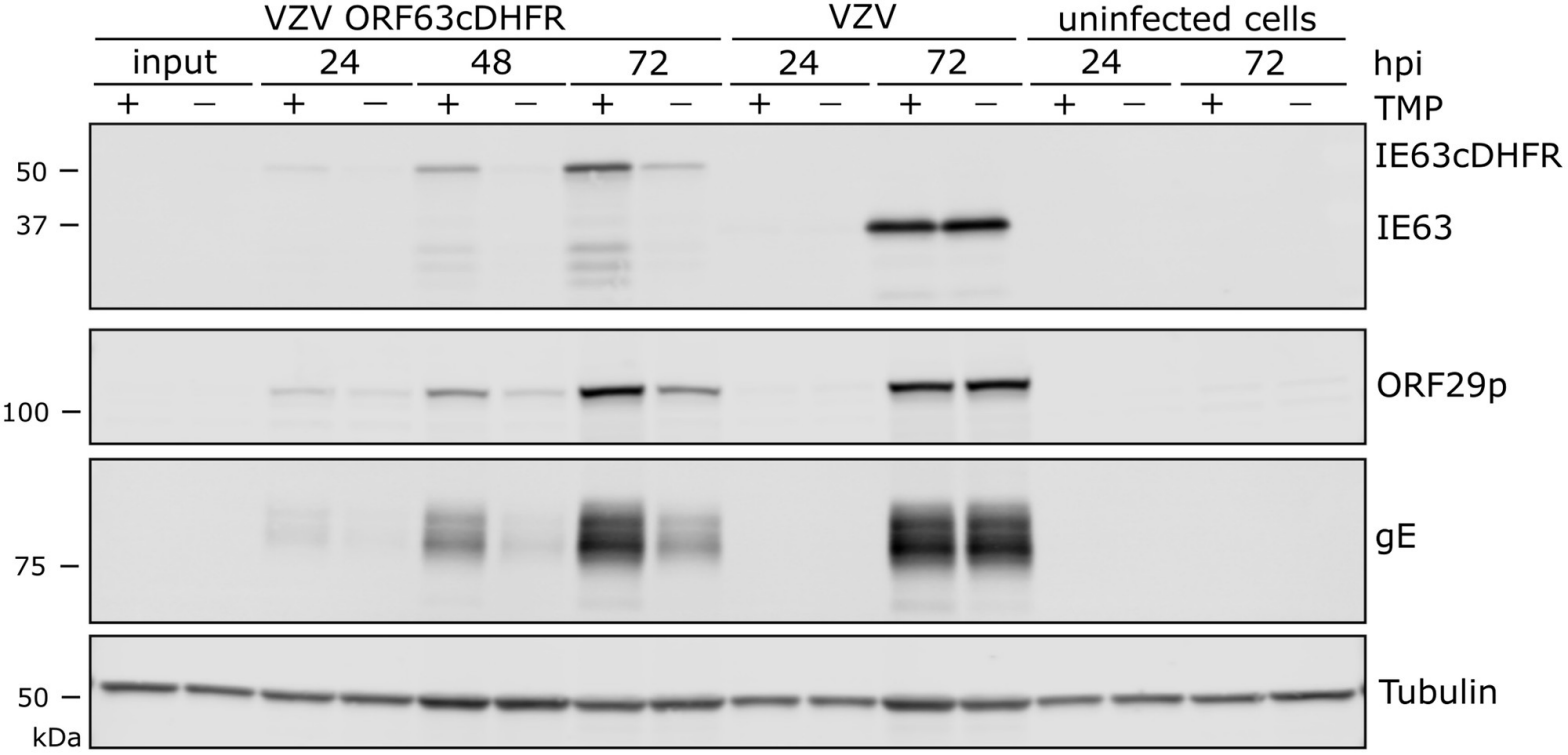
VZV IE63 is regulated by DHFR degron domain fused to carboxy-terminus. Proteins were analyzed similarly to that shown in Figs 3 and 4. Protein samples were of cells infected with VZV ORF63cDHFR, pOka, or uninfected cell equivalents up to 72-hpi. Wild-type ORF63 (IE63) and DHFR modified (IE63cDHFRp) protein, proteins from alternative kinetic classes (ORF29p, gE), or cellular control (alpha-tubulin) were compared under TMP permissive (+) and nonpermissive (-) conditions. The blots are scanned in linear range and representative of two identical experiments.

We next assessed protein production by the three conditional viruses over a growth period of 72-h, in the presence or absence of TMP. Infections were initiated at low multiplicity (1 infected to 100 uninfected cells) so that multiple rounds of VZV infection could occur. For VZV ORF9cDHFR infections, protein levels made in the continued presence of TMP were similar to that made by wild-type VZV (pOka) (Fig 3), but in the absence of TMP, VZV protein accumulation was dramatically reduced in VZV ORF9cDHFR infections. ORF9cDHFRp showed the expected size increase of about 18 kDa as a result of degron addition, and almost no unfused ORF9 protein in VZV ORF9cDHFR infections was detected. Of note, multiple forms of ORF9p and ORF9cDHFRp were detected, consistent with previous reports of this being a phosphoprotein [67]. For infections initiated with VZV ORF4nDHFR, the expected size increase due to the addition of the degron was apparent, and TMP-dependent protein production increased over a 72-h infection in contrast to pOka, but little in the absence of TMP (Fig 4). VZV proteins from ORF29p and gE were greatly reduced in VZV ORF4nDHFR infections grown in the absence of TMP, consistent with impaired viral spread and amplification, and the TMP-dependent loss of infectivity in growth curves (Fig 2B). Infections initiated with VZV ORF63cDHFR resulted in expression of the larger IE63cDHFR in the presence of TMP as expected (Fig 5), and it was the predominant form made by the recombinant virus. Reduced levels of protein accumulated under nonpermissive conditions, although some accumulation of IE63cDHFR occurred in the absence of TMP over time. The increase in accumulation of other VZV proteins is consistent with a slightly leaky phenotype for this virus.

**Fig 6:**
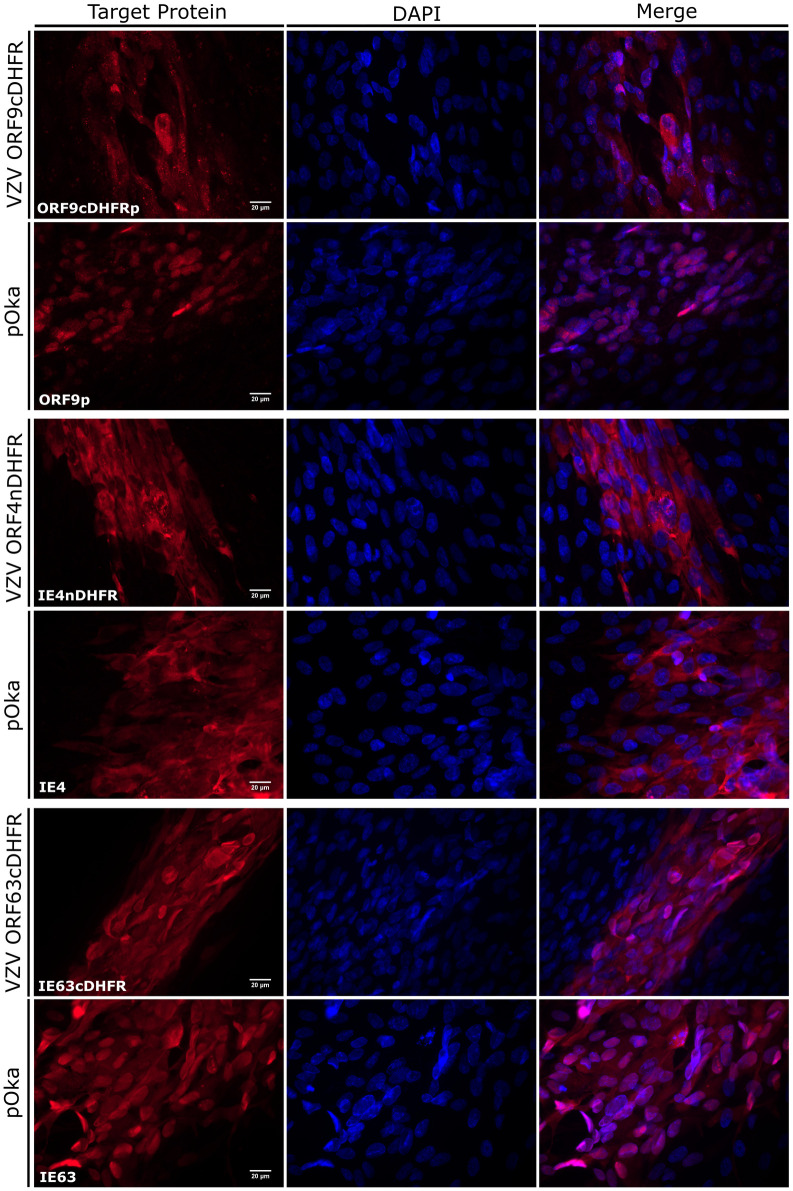
Localization of degron fused viral proteins compared to wild-type VZV proteins in infected cells. (A) Images show the edges or small regions of plaques formed by wild-type VZV (pOka), VZV ORF9cDHFR (top), VZV ORF4nDHFR (middle), or VZV ORF63cDHFR (bottom) at 2-dpi. Protein cellular distribution in fixed cells was imaged to represent the distribution seen in the cultures after staining with antibodies to ORF9p, IE4, or IE63. The ‘target protein’ column indicates the specific protein probed, as noted in the lower left corner of the column. The center column shows DAPI stained nuclei, and the rightmost column shows a merged panel of target protein immunofluorescence and DAPI staining. Magnification: 60X (N.A. 1.25) oil. Single images are representations of a minimum of 15 images analyzed for each virus.

Further characterizations were carried out to address if the degron addition had an influence on subcellular localization of the fused proteins. The nuclear localization (NLS) and/or nuclear export signals (NES) have been previously located for each protein [65,70–72]. The cellular localization of IE4nDHFR, ORF9cDHFRp, and IE63cDHFR were compared to the unmodified proteins made by wild-type pOka infected cells, all grown in the presence of TMP. Images were acquired from edges of 2-dpi individual plaques showed that, in general, protein localization for each DHFR fused protein was similar to the distribution of the native proteins (Fig 6). IE4 and IE63 from pOka localized to both nuclear and cytoplasmic compartments, with IE4 showing a more predominantly cytoplasmic distribution in most cells. IE63 was more nuclear localized in cells at the edge of plaques, which represent earlier stages of infection as compared to distributions at plaque centers. ORF9p was seen in both nuclear and cytoplasmic compartments in both DHFR and wild-type VZV infected cells. The similar distribution of the DHFR and wild-type proteins for each virus suggests that degron addition does not greatly affects subcellular distribution of the proteins.

We next sought to determine if these genes were expressed at detectable levels in DRG of inoculated rats. Previous studies have found that VZV transcripts [25,73] and proteins [26,28,30] can be detected by *in situ* hybridization or immunohistochemistry in ganglia after inoculation. We used a set of described PCR primer/probes to detect ORF62 and ORF63 [56], and additional sets to identify ORF4 and the DHFR degron domain by RT-qPCR. The TMP-dependent virus stocks were generated as detailed in the methods and were the same used throughout all animal studies. Titrated virus was inoculated into rat footpads. The L4, L5, and L6 DRG of animals harvested at days 4-, 5-, and 7-dpi was used to prepare total RNA and then converted to cDNA for assessment of VZV gene expression in DRG (S3 Fig). We were unable to obtain statistically significant increases in gene expression for the probed VZV genes. This indicates that levels of transcripts for ORF62, ORF4, and ORF63, or the DHFR sequence are too low for consistent detection by this approach in rats infected with wild-type VZV, VZV ORF4nDHFR, and VZV ORF63cDHFR. The lack of significance when compared to relative changes in GAPDH is similar to our previous study [32] and is generally consistent with sparse detection of VZV transcripts by *in situ* hybridization studies. Despite low detection levels for VZV ORF63cDHFR infected rats, the similar pattern that formed in the detection of ORF63 and DHFR, which should identify the same transcript, may suggest these data are consistent with the ORF63 transcript being detectable in rat ganglia at low levels [25].

### Analyses of rats inoculated with VZV ORF9cDHFR indicate productive replication is not required for VZV-induced chronic hypersensitivity behaviors in rats

**Fig 7:**
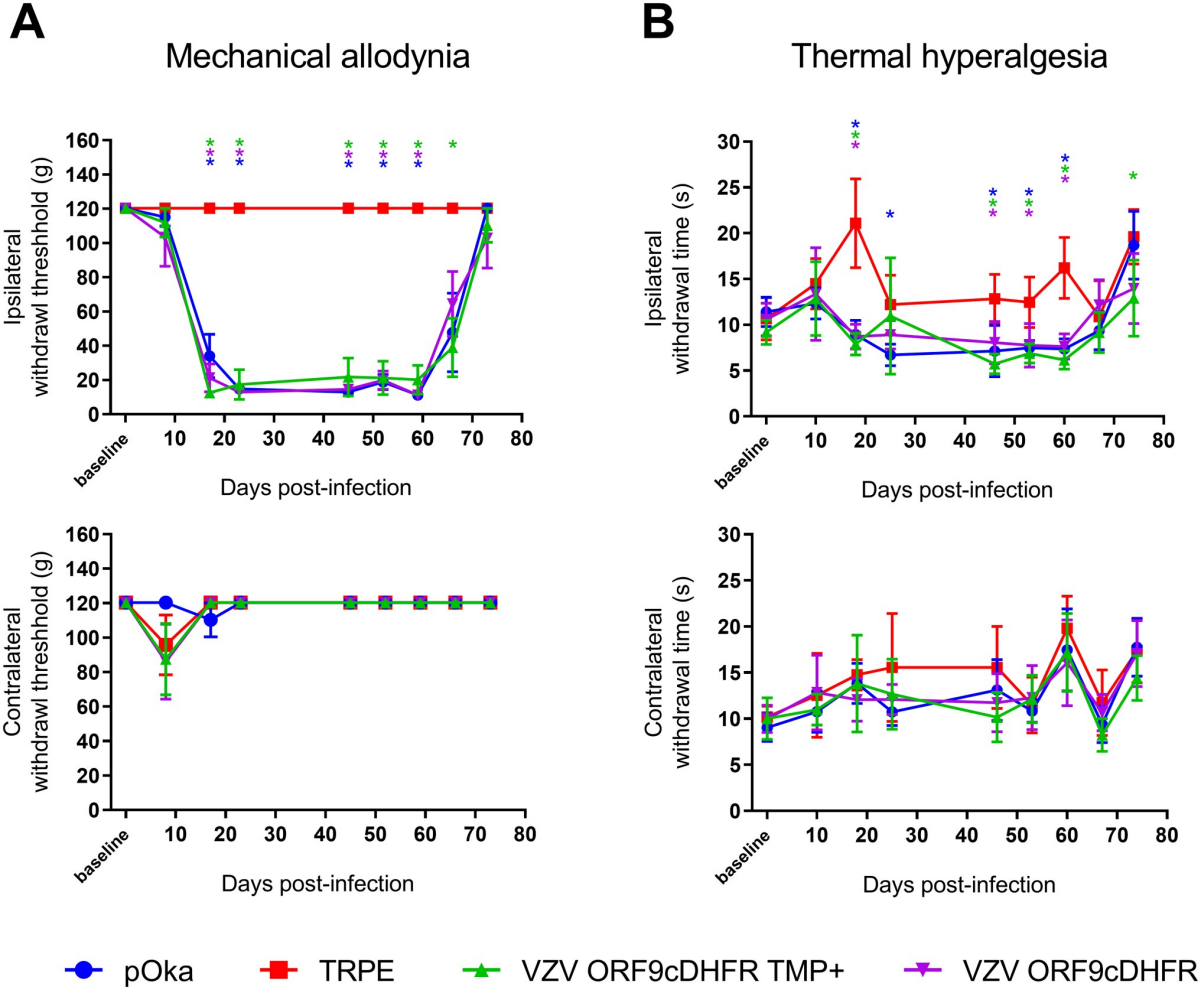
Development of mechanical and thermal hypersensitivity in rats after VZV ORF9cDHFR footpad inoculation under permissive and nonpermissive growth conditions. Male Sprague-Dawley rats (n = 6/group) were acclimated to measurement conditions and a baseline response was established the day of inoculation. Rats were inoculated in one rear footpad with 2x10^5^ PFU TRPE-associated pOka (blue circle), VZV ORF9cDHFR in PBS with 500 nM TMP (green triangle) or without TMP (purple triangle), or uninfected TRPE cell equivalent (red square). Mechanical hypersensitivity (A) was measured by von Frey monofilaments and the Up-Down method. MA assessment of the inoculated footpad was measured as 50% withdrawal threshold in grams (g) in the inoculated footpad (top) and the contralateral, uninoculated footpad (bottom) of the same rats. Thermal hypersensitivity (B) was assessed by Hargreaves apparatus as detailed in the methods and is presented as time-to-withdrawal post-light activation (seconds). The responses of the inoculated footpad (top) and contralateral footpad of the same rats (bottom) is shown. Error bars: SD. Statistics: Two-way ANOVA with Bonferroni multiple comparison to uninfected cell control, where * = p < .05 and is color coded by group.

We next used the conditionally replicating VZV to assess how reducing viral gene expression and replication influenced VZV induction of prolonged nocifensive behaviors in rat models of PHN. All TMP-dependent VZV were generated in cells supplemented with TMP. All viruses were managed identically for consistency, with the TMP-supplemented inoculant resuspended in PBS containing 500 nM TMP instead of PBS alone. Animals were then inoculated into the rear footpad as detailed previously [31], in a random left/right manner (n = 6) so the inoculated paw and the nature of the inoculant was blinded from the behavioral assessors. Rat groups received equivalent infectious units of VZV ORF9cDHFR containing 500 nM TMP, the same virus without TMP, wild-type pOka as the positive control, or uninfected cell equivalent as the negative control. Development of mechanical allodynia (MA) and thermal hyperalgesia (TH) was assessed over a period 74 days (Fig 7). As predicted, pOka inoculated animals developed mechanical hypersensitivity responses by day 14, consistent with previous studies [26–32]. Hypersensitivity was significant at multiple time points when compared to the contralateral (uninfected) footpad or rats that received uninfected cells, which all developed no significant hypersensitivity over the course of the study. Importantly, VZV ORF9cDHFR induced significant hypersensitive states lasting over the testing period similar to that seen for pOka inoculated animals, whether or not a bolus of TMP was administered with the virus in the inoculate. Mechanical responses lasted several weeks (Fig 7A) and began to wane towards the end of this study, so that hypersensitive responses at 66-dpi were no longer significant for the pOka and VZV ORF9cDHFR groups compared to controls. Measurements of thermal hypersensitivity followed a similar pattern, though the withdrawal time difference between positive and negative thermal responses was more subtle, so that significance in the differences was not seen for every time point. By 18 dpi, significant differences were detected in all VZV inoculated groups when compared to the uninfected cell group or contralateral paw measurements (Fig 7B). The variability in thermal response is consistent with previous studies [26,28,29,31]. An average withdrawal time under 7-seconds was observed in hypersensitive rats, while responses for negative control animals remained above 10-seconds throughout most measurements in the 74-day study. Thermal hypersensitivity also waned during the later stages of the study. Given that VZV ORF9cDHFR replication is TMP-dependent and would be unable to spread when inoculated in the absence of TMP, we take these results to indicate that the development of hypersensitivity behaviors in the rat footpad model does not require productive replication, but is more the result of a single round, non-productive infection after inoculation.

**Fig 8:**
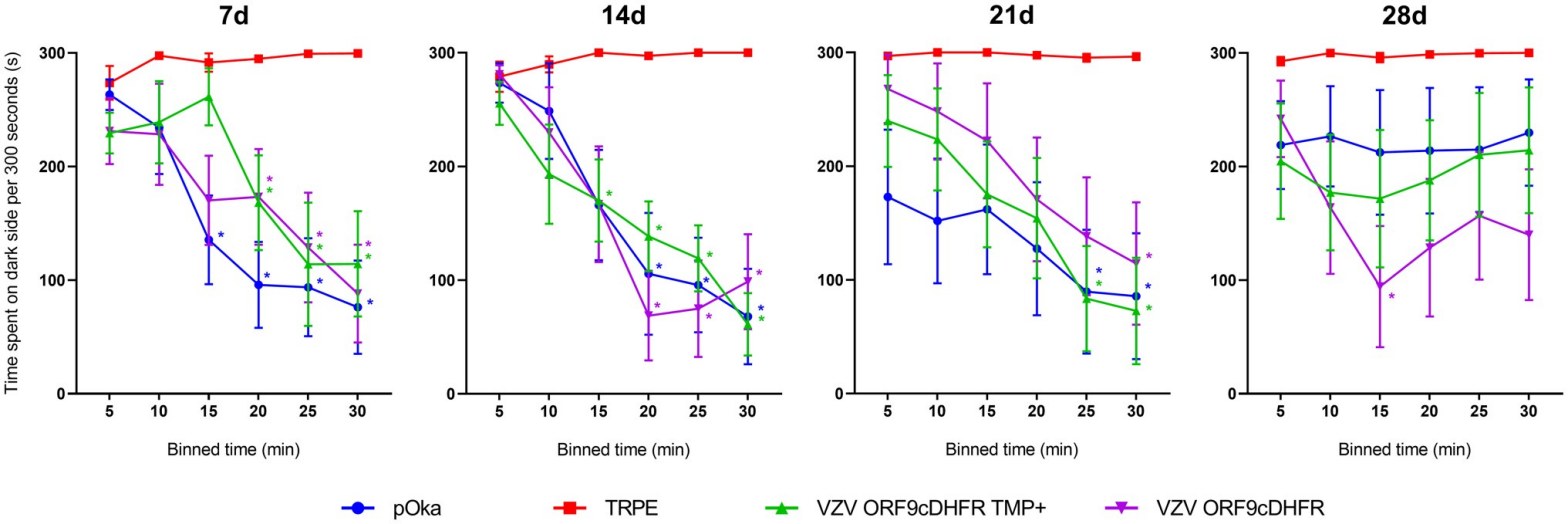
Affective pain develops in rat whisker pad following inoculation with replication conditional VZV ORF9cDHFR under both growth-permissive and nonpermissive conditions. Male Sprague-Dawley rats (n = 7/group) received inoculation into the whisker pad of 2x10^5^ PFU TRPE-associated pOka (blue circle), VZV ORF9cDHFR in buffer with 500 nM TMP (green triangle) or without TMP (purple triangle), or uninfected TRPE cell equivalent without TMP (red square). Hypersensitivity was measured at the times indicated by days (d) post-infection above each graph using a PEAP method detailed in methods. Time spent in the dark side (y-axis) of the enclosures is shown after repeated stimulation with a 60g (5.88) von Frey hair every 15 seconds, assessed over 5-min bin periods for a total of 30 min (x-axis). The side of the face stimulated depended on the position of the rat’s head, with facial stimulation at the inoculated side if its head is in the dark side of the enclosure, and stimulation of the uninoculated side of the face if in the light side of the enclosure. Error bars: SEM. Statistics: Two-way ANOVA with Bonferroni multiple comparison to TRPE negative control where *p < .05 and color coded by group.

We also evaluated the ability of these viruses to induce affective pain responses in the rat facial model (Fig 8). Rats were inoculated at the whisker pad in the same groups as the footpad experiments, and rats were assessed for nocifensive behaviors in a fully blinded manner using the Fuchs’ PEAP assay [34,60]. This physiological test evaluates animal behaviors resulting from noxious stimuli, in which rats show reluctance to locate to preferred locations if the stimulus evokes higher levels of pain or sensitivity. Animals receiving uninfected cells showed no behavioral indicators of aversion to stimulation of the inoculated whisker pad and remained predominantly on the dark or preferred side of the enclosure over the course of the experiment. However, stimulated animals that received pOka showed a considerable reduction of time spent on the dark side of the enclosure. Such behaviors began to return to baseline, by 28-dpi and were no longer significant. While VZV-induced affective pain indicators at the whisker pad were shorter-lasting than the mechanical and thermal hypersensitivities detected at the footpad, the behavior of rats inoculated at the whisker pad with different viruses were consistent with the footpad data, in that VZV ORF9cDHFR induced hypersensitivity responses when inoculated with or without TMP. These data support the hypothesis that VZV ORF9cDHFR retains the ability to induce hypersensitivity with or without conditional replication and is consistent with the conclusion that ongoing replication of VZV is not required for the induction of pain behaviors.

### Analyses of VZV ORF4nDHFR indicates production of the VZV IE4 is required for development of VZV-induced hypersensitivity

**Fig 9:**
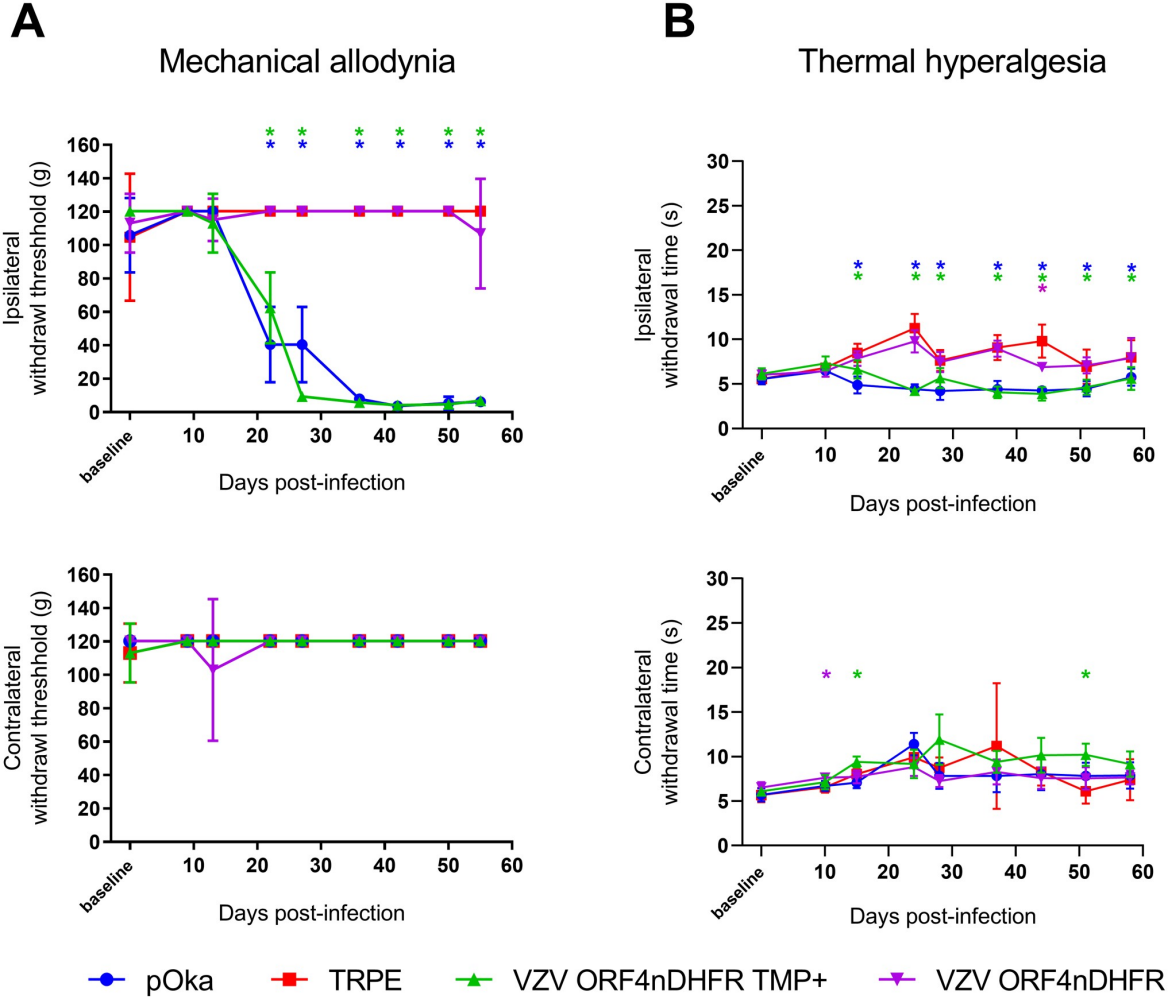
Footpad inoculation by VZV ORF4nDHFR induces mechanical and thermal hypersensitivity only if IE4 is stabilized under growth-permissive conditions. Male Sprague-Dawley rats (n = 6/group) were inoculated with 2x10^5^ PFU pOka (blue circle), VZV ORF4nDHFR with 500 nM TMP (green triangle) or without TMP (purple triangle), or uninfected TRPE equivalents (red square). (A) MA of inoculated footpad (top) vs contralateral (bottom). (B) The same group of rats were tested for TH of the inoculated footpad (top) vs contralateral, uninoculated footpad (bottom). Error bars: SD. Statistics: Two-way ANOVA with Bonferroni multiple comparison to TRPE negative control where *p < .05 and color coded by group.

A similar set of studies were performed in the footpad and facial models to examine how VZV ORF4nDHFR stimulates hypersensitivity when replication is permitted or prohibited. In footpad model studies (Fig 9), responses indicating mechanical hypersensitivity developed by 22-dpi in pOka inoculated rats. The timing of onset of hypersensitivity was later than that seen in the ORF9cDHFR study, but such variability has been seen previously [26,28,31,32]. No measurable hypersensitivity developed in rat footpads injected with uninfected cells or developed in the contralateral uninoculated footpads. In contrast to the results obtained from animals inoculated with VZV ORF9cDHFR, hypersensitivity developed in rats that received VZV ORF4nDHFR supplemented with 500 nM TMP, but animals did not develop hypersensitivity if TMP was not included in the inoculum. Rather, animals showed withdrawal responses similar to uninfected cell equivalent groups (Fig 9A). Hypersensitivity in animals inoculated with VZV ORF4nDHFR with TMP was detected throughout the entirety of the assessment period (the study was terminated at 55 dpi). Thermal hypersensitivity responses followed a similar pattern (Fig 9B) so that by 24-dpi, significant hypersensitivity was seen at select times in rat groups that received pOka or VZV ORF4nDHFR containing TMP. Quicker withdrawal times did not develop in contralateral footpads, footpads inoculated with uninfected cells, or in footpads that received VZV ORF4nDHFR without TMP supplementation. Rat groups that developed hypersensitivity remained significant at most measurement times until to the end of the experiment when compared to the negative control. We did see some significant responses in the contralateral footpad at certain timepoints, but this is not unusual and has been observed at random and on occasion in previous thermal hypersensitivity assessments. However, these contralateral measurements did not form a consistent pattern of continued hypersensitivity, as observed on the ipsilateral side, and generally resembled the responses of the negative control group.

**Fig 10:**
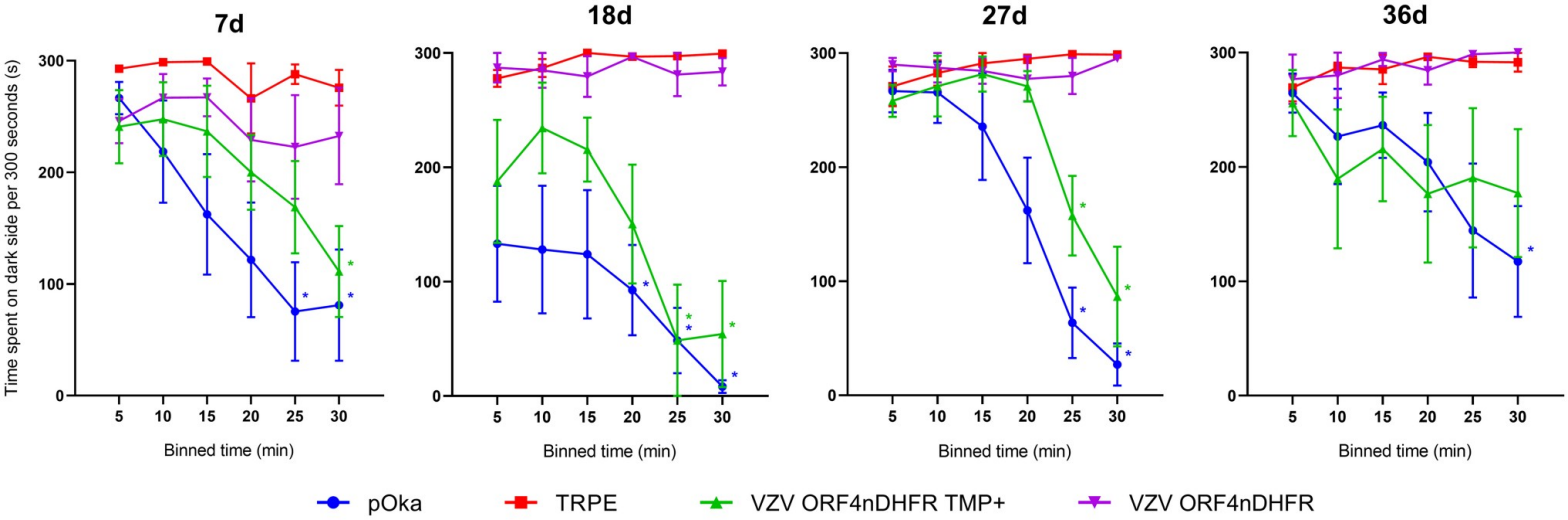
Affective pain develops following whisker pad inoculation with replication conditional VZV ORF4nDHFR only under permissive conditions. Male Sprague-Dawley rats (n = 6/group) received 2x10^5^ PFU pOka (blue circle), VZV ORF4nDHFR with 500 nM TMP (green triangle) or without (purple triangle), or uninfected TRPE cell equivalent (red square). Hypersensitivity was measured at times post-infection (d) indicated above each graph using the same methods as Fig 7 and detailed in the methods section. Error bars: SEM. Statistics: Two-way ANOVA with Bonferroni multiple comparison to TRPE negative control where *p < .05.

In the rat facial model of affective pain (Fig 10) the pOka and VZV ORF4nDHFR with TMP groups were found to spend significantly more time on the light side of the enclosure at 7-dpi when compared to the uninfected cell equivalent group. Consistent with the footpad studies, animals that received VZV ORF4nDHFR without TMP continued to spend a majority of time on the dark side as seen for the uninfected cell group. The trend continued over the course of the 5-week experiment, at which time nocifensive behaviors of the hypersensitive groups waned, as seen previously [34]. Post-hoc analysis indicates significance for the pOka group during all measurement groups, while the VZV ORF4nDHFR TMP supplemented group lost significance during the final measurement timepoint. At no point did the VZV ORF4nDHFR without TMP group show any significant indication of hypersensitivity. We highlight that these results contrast with the responses of animals receiving VZV ORF9cDHFR, in which pain behaviors developed with or without TMP. The results suggest that the degron mediated removal of IE4 prevents the development of pain behaviors and suggests that IE4 is necessary for the induction of pain responses after VZV inoculation.

### Analyses of additional VZV mutants confirms the requirement for gene expression, but not full viral replication for the development of pain behaviors in rats

**Fig 11:**
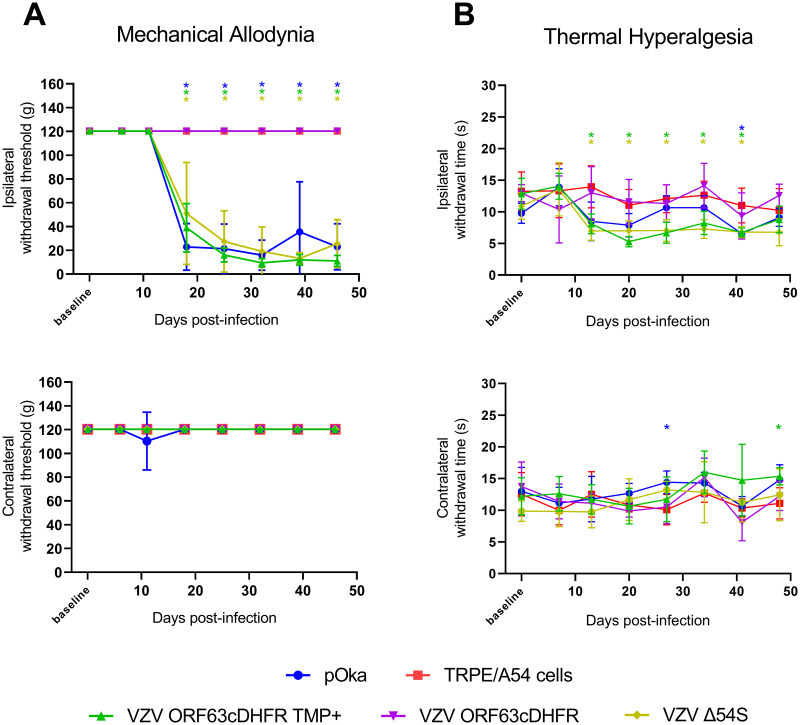
Induction of mechanical and thermal hypersensitivity by VZV with conditionally stabilized IE63 or by VZV lacking the production of the ORF54 portal protein. Male Sprague-Dawley rats (n = 6/group) were inoculated with 2x10^5^ PFU pOka (blue circle), VZV ORF63cDHFR with 500 nM TMP (green triangle) or without TMP (purple triangle), VZV Δ54S (yellow diamond), or uninfected cell equivalents (red square) and subjected to the same testing methods in Fig 6. (A) Mechanical hypersensitivity of inoculated footpad (top) vs contralateral (bottom). (B) The same group of rats were tested for thermal hypersensitivity of inoculated footpad (top) vs contralateral (bottom). Error bars: SD. Statistics: Two-way ANOVA with Bonferroni multiple comparison to TRPE negative control where *p < .05. Note: The uninfected cell equivalents (red square) in this figure represent inoculated rats n = 3 A54 cells and n = 3 TRPE cells. This data has been combined for clarity and was deemed appropriate as the cell lines are from a common lineage.

We sought to confirm the contrasting outcomes of the VZV ORF9cDHFR and ORF4nDHFR in the rat footpad model through the analyses of additional VZV recombinants containing mutations in different genes. We evaluated animals inoculated with VZV ORF63cDHFR in the same manner (Fig 5). In parallel, we examined the responses in the footpad model of animals that were inoculated with a recently described recombinant VZV that is deleted for expression of ORF54 (VZV Δ54S) [45]. ORF54 encodes the portal protein involved in the packaging of viral DNA into preassembled capsids in the nucleus, and VZV lacking ORF54 cannot replicate beyond the initial round of replication in non-complementing cells. To grow VZV Δ54S, an ARPE-19 based complementing cell line was used (A54). Following footpad inoculation, the behavioral responses of rats receiving these viruses and controls were assessed for mechanical (Fig 11A) and thermal hypersensitivities (Fig 11B). In these studies, the negative control group was divided into two, with one group of rats inoculated with uninfected A54 (n = 3) and the second with uninfected TRPE (n = 3) cells. The behavioral responses of the two uninfected cell controls were indistinguishable from each other (and thus combined in the graph) or historical uninfected cell controls, indicating that the complementing cell line did not induce a significant pain response. pOka infected cell inoculated animals developed significant mechanical hypersensitivities from 18-dpi onwards that lasted through to the end of the study (Fig 11A). Thermal hypersensitivities for pOka inoculated animals also trended towards a response, although the measurements in this study were not significant at times other than 40 days (Fig 11B). Animals that received VZV ORF63cDHFR with a bolus of TMP at the footpad developed significant mechanical hypersensitivity at a time similar to those animals receiving pOka, but animals receiving the virus without TMP did not develop any significant hypersensitivity behaviors and showed responses similar to those of animals receiving uninfected controls at all timepoints. This result indicates that the production of IE63 is critical for the induction of a prolonged mechanical hypersensitivity response in the rat model. In contrast, significant and long-lasting mechanical pain responses developed in animals inoculated with the genetic mutant VZV Δ54S. While this virus was administered with its complementing cells, the subsequent infection of any cells in the rat host would not be expected to progress to infectious virus production. These studies further support our previous conclusions involving VZV ORF9cDHFR (Figs 7 and 8) and ORF4nDHFR (Figs 9 and 10) and denote that hypersensitivity in the rat models of PHN require early infectious processes and the expression of regulatory proteins, but that this occurs during a single and abortive round of infection in the rat host.

## Discussion

In this work, we developed and exploited novel VZV mutants to dissect how components of the VZV infectious process contribute to the development of nocifensive behaviors in rat models of PHN. The data establish that presence of two VZV regulatory proteins, IE4 and IE63, are required for the development of hypersensitivity, but that production of infectious progeny virus in cells of the rat host is dispensable. The results imply that development of hypersensitivities in rats inoculated with VZV are the consequence of a single round, abortive infection in cells of the rat host but require some level of viral gene expression. These data also suggest that hypersensitivities are not the result of a reaction (immune or otherwise) to the VZV-associated cell antigens injected but require VZV to initiate an infection in cells of the rat host. The studies have implications for the induction of hypersensitivity and PHN in humans: we speculate that the partial VZV expression program could occur in human ganglia after an HZ event, in which infected but surviving neurons have a destabilized host neuronal homeostasis that leads to prolonged signaling of pain that may underlie PHN.

This is the first report of conditionally replicating VZV mutants, and was achieved by adopting a protein degron system that had been developed to study the consequences of protein turnover and removal in eukaryotic cells [39]. Viable VZV with ORFs 4, 9, or 63 containing the 160-amino acid degron were TMP growth regulated, so the degron addition had minimal overall effect on the function of the targeted proteins. Each virus showed similar levels of growth and protein production over time compared to wild-type VZV in growth-permissive conditions and formed plaque sizes that were only slightly reduced under permissive conditions. The degron did not strongly influence intracellular localization of the fused proteins, consistent with only a modest, if any influence on protein function. In the absence of TMP, each virus showed severely limited capacity in growth, viral spread, and protein production, with ORF4 and 9 degron viruses showing tighter regulation than VZV ORF63cDHFR. This system was previously applied to study of the role of SVV ORF63 [64]. The degron system could clearly be useful to evaluate additional VZV essential genes. It has the advantage of permitting growth of mutant viruses in any permissible cell type. It also circumvents the need to derive complementing cell lines expressing the VZV gene-of-interest *in trans* in order to propagate VZV with a deleted gene, which has generally appeared to be more difficult for VZV in the limited human cell lines that support its growth. Previously developed VZV lacking ORF9 [47] and ORF4 [63] were grown by complementing the absent gene in cells that had been transduced with high-titer baculoviruses expressing a cytomegalovirus IE promoter driven VZV gene, which then required treatment with sodium butyrate to inhibit type-1 histone deacetylase activities and the chromatin-mediated silencing of the baculovirus. We attempted to prepare the VZVΔORF4 virus detailed previously [63], but could not obtain titers sufficient to exploit in the rat PHN models. The baculovirus approach is also complicated by the potential of the baculovirus itself and the sodium butyrate treatment required for transgene expression to affect behavioral responses [74–76]. The lack of success discouraged our pursuit of the VZVΔORF9 virus detailed previously [47]. VZV lacking the duplicated ORF63 and ORF70 has been reported by one group to replicate without complementation [77], but others report that deletion of ORF63 and ORF70 abrogated virus growth [78,79]. As far as we are aware, no ORF63 complementing cell line or system has been described. However, a caveat is that the conditional replication strategy is unlikely to be applicable to all VZV genes. Our attempts to target three DNA replication proteins did not result in viable virus (Table 2), suggesting that the degron addition interfered with essential protein functions. We also found that degron addition may not regulate protein stability, as found for VZV containing the degron added to the major transcriptional regulator encoded by ORF62 and ORF71. The reason for a lack of regulation is not clear, but it could be due to accessibility of the degron tag to ubiquitin-ligases as a consequence of cellular compartmentalization. For such genes, VZV deletion virus may require more classic complementation methods, and there has been some recent success in developing ARPE-19 based cell lines such as used here to grow the VZV Δ54S [45]. We are currently extending the degron system to evaluate additional VZV genes involved in DNA replication, to ask if VZV blocked at the DNA replication stage are able to induce hypersensitivity responses.

The data show that productive VZV replication in the rat PHN model is not required to cause VZV-induced pain in those models. This fits with our suspicion that the high species specificity of VZV prevents viral replication *in vivo* at some post-entry phase in rats. While rats have long been used as both models of pain and as models of latency, VZV permissivity in the rat has never been fully resolved. Numerous studies report the detection of viral transcripts and some proteins in VZV infected rat ganglia that were hypothesized to reflect the VZV latent state [25,26,28,32,73,80,81]. The long-term pain behaviors have been useful to examine potential pain alleviating drugs [26–29,31,82,83]. Dalziel *et al*. (2004) found that that pain induced by VZV infection was not alleviated by a 10-day treatment with systemic acyclovir (ACV) administration to rats [27]. ACV inhibits herpesvirus DNA replication, and blocked pain responses generated in rats receiving HSV, suggesting these two related herpesviruses induced pain by different mechanisms. Our work showed that UV-irradiation of the VZV infected cell inoculate (to reduce infectivity by more than 2-logs) prevented development of most pain behaviors [31]. However, we reasoned that neither approach was definitive in determining if VZV replication was required for induction of pain behaviors. The minimum inhibitory concentration of ACV for VZV is considerably higher than for HSV and the results of Dalziel *et al*. may have reflected insufficient levels to block VZV. UV-irradiation may have caused considerable damage to the inoculum. However, our finding that two mutants unable to fully replicate under our inoculation conditions (VZV ORF9cDHFR and VZV Δ54S) allows us to conclude that potential ongoing VZV productive replication, should it occur in rats, is not needed for development of nocifensive behaviors. Both mutants would likely be blocked at late stages of infection and express most VZV genes; ORF9p is essential and primarily involved in tegument assembly and secondary envelopment [47,67], while VZV lacking ORF54 would not be able to package DNA into capsids. Though VZV Δ54S was administered in complementing cells, the virus produced would be unable to form assembled virions in cells of the rat. A54 cells induced responses that were similar to uninfected cells in the same experiment and in historical studies [30–34]. Thus, the rat pain models appear to reflect a nonpermissive host in which abortive infection by VZV is sufficient for pain indicators.

In contrast, studies with the ORF4 and ORF63 conditionally replicating VZV establish that some VZV gene expression is essential for the induction of mechanical hypersensitivities. ORF4 is expressed as an IE gene based on classic cycloheximide-actinomycin D reversal experiments after cell-free VZV infections [41,68,69]. The protein has post-transcriptional regulatory activities suspected to be involved in nuclear export of intronless mRNA, in a manner similar to HSV ICP27 [84]. Removal of this protein from the infectious process would likely severely limit downstream VZV gene expression programs, as most VZV lytic transcripts are not spliced. Similarly, ORF63 was shown to be IE expressed using cycloheximide-actinomycin D approaches, and studies indicate it is a regulatory protein that is critically involved in early infectious processes [42]. Rats inoculated under conditions of TMP-permitted replication with each virus developed long-lasting hypersensitivities, establishing that the viruses themselves were not defective, but under nonpermissive replication conditions, neither induced significant behavioral responses. The data solidifies that some VZV genes and proteins with regulatory function are needed in rats for prolonged pain indicators. Presumably, VZV lacking IE4 would not shuttle intronless VZV messenger RNA to the cytoplasm for translation [69]. What the consequences of ORF63 are on the rest of the VZV expression program is less clear. The lack of any response without permission to replicate also indicates that antigen load in the inoculate, and components contained in the infecting virus tegument and capsid, are not sufficient to drive the signaling processes associated with hypersensitivity.

At this stage, there are some questions that remain to be resolved. We do not yet know whether the production of IE4 and/or IE63 are the actual drivers of the pain responses, or if their roles are to permit the expression of downstream genes that induce nocifensive behaviors. The resolution of this would require the study of additional mutants, such as a mutant that would enable us to prevent DNA replication in the rat host. Based on our previous work, where we did not see DNA replication in primary rat cell cultures, we would predict that a VZV with conditional degron-controlled essential DNA replication protein might show the same type of hypersensitivity induction seen for VZV ORF9cDHFR and induce hypersensitivity without DNA replication. Such mutants are being developed. It is also not yet clear as to what tissues the VZV limited expression program is needed to induce the hypersensitivity responses. We hypothesized that VZV proteins are produced within a few neurons of a sensory ganglion that innervate the site of inoculation, and that these trigger altered neuronal signaling. However, studies to determine significantly increased expression of VZV transcripts in rat ganglia have not been successful here and in a previous report beyond 5–7 days [32]. This indicates that VZV gene expression within innervating neurons is low and could be transient. Others have reported the detection of IE62 and IE63 in ganglia obtained after *in vivo* inoculation at sparse levels [24–26,73] and gene array studies suggest there are some changes in the respective ganglia [32]. We postulate that further studies of transcripts at the single cell level of the ganglia may allow the correlation of host with viral gene expression, but we considered them outside of the scope of the current work. It is even possible that the essential components of VZV gene expression is in non-neuronal cell types, such as glia and support cells proximal the sensory nerve endings at the periphery. These studies are planned or in progress.

Taken together, the data are consistent with the hypothesis that a partial gene expression program is required and sufficient for the induction of hypersensitivity in rat models of PHN. We speculate that this may be quite relevant to clinical HZ and the development of PHN, despite the fact that PHN follows reactivation whereas the rat model reflects events after a primary inoculation. However, when VZV reactivates in sensory ganglia, it probably starts within one or a small fraction of neurons, usually within a single ganglion. VZV then undergoes intraganglionic spread by cell-cell fusion, spreading to other neurons that are, therefore, newly infected [85,86]. These deliver virus to the periphery via innervating axons that terminate throughout the dermatome. There is neuropathic damage in many neurons and a reduction of nerve fibers in the afflicted dermatome [13]. Ganglionitis has been proposed to partly account for acute pain associated with HZ [18]. However, the ganglionic replication of VZV is usually limited, probably because of intrinsic, innate, and VZV-specific adaptive immune responses. We know that in HSV ganglionic infection models, ganglia resident CD8+ T cells limit virus gene expression and can suppress active neuronal replication through non-cytolytic means [87,88]. As such, the neuron survives, despite having initiated some viral gene expression. We propose this occurs on a much larger scale in the human ganglion hosting the HZ reactivation event, and clinical PHN may reflect the activity of neurons that have been infected, have made some VZV proteins (that induce host cellular changes) but then become subjected to multiple noncytolytic effectors, such as mediated by ganglion resident VZV-specific T cells that halt the full infectious program in a non-cytolytic manner. We postulate that a limited viral protein expression program (and/or the immune effectors targeting them) in surviving neurons may undergo altered host gene expression programs that involve genes of pain signaling. We are currently examining how individual VZV gene products may induce development of PHN-like behaviors in the rat models and the host expression programs that result. This may identify mechanisms that can be therapeutically targeted to limit the development of PHN.

## Supporting information

S1 Table Primers for RT-qPCR. TaqMan primer/probe sets used in RT-qPCR analysis of VZV-infected rat DRG. 6-FAM (6-carboxyfluorescein). BHQ1 (black hole quencher 1).
(DOCX)

S1 Fig *Kpn*I restriction enzyme analysis of viral nucleocapsid DNA and Southern blot with DHFR-specific probe to show expected insertion sites and VZV ORF63cDHFR homologous recombination to ORF70 within the TR_s_ region. Southern blots (left) are aligned with the ethidium bromide-stained 1% agarose gel electrophoresis image of separated fragments following *Kpn*I digestion of purified VZV nucleocapsid DNA from each DHFR degron inserted virus, or from virus derived from the parental VZV BAC (right). The DHFR probe generated by PCR predominantly hybridized to the same large DNA gel fragment for VZV ORF9cDHFR and ORF4nDHFR. For VZV ORF63cDHFR, two DNA fragments seen in other viruses (and not hybridizing the DHFR probe) increased in size by 480-bp and both hybridized the DHFR probe, with the larger representing ORF63 and smaller representing ORF70, respectively. A map of the regions of the DNAs for the expected *Kpn*I DNA fragments in each virus is shown below the gel images with the expected fragment size indicated with and without the DHFR sequence insertion. The green vertical bar in the lower diagram for VZV ORF63cDHFR represents the position of the insertion of the BAC mini-F sequence (~8 kb) that self-excises with virus derivation and passage. A minor low abundance DNA fragment hybridizing the DHFR probe of ~6000-bp in size is present in every virus and was judged to be due to non-specific hybridization. Southern blot images were acquired on LICOR Odyssey in linear range. Created with BioRender.com.
(TIF)

S2 Fig *Sph*I restriction digestion and Southern blot analysis to show DHFR degron insertion. Southern blotting of 1% agarose DNA-separated *Sph*I digested fragments with a DHFR-specific probe (left) and the ethidium bromide-stained VZV nucleocapsid DNA after gel electrophoresis (right). A map of the DNA fragments is shown at the bottom for each virus DNA as predicted from insertion at the correct sites for each virus. The map shows the predicted DNA fragment size with and without the degron sequence insertion. The sizes of a DNA ladder are shown in the composite image. The blots reveal that the degron insertions for each virus result in the increase of specific DNA fragment by 480-bp that then are the main fragments hybridizing the DHFR probe as predicted. Two ~6000 bp fragments hybridizing the DHFR probe at low levels for VZV ORF4nDHFR DNA are of sizes expected from partial digestion products at low levels in which the expected fragment is not restriction digested from the adjacent *Sph*I DNA fragment. Created with BioRender.com.
(TIF)

S3 Fig Detection of ORF4, ORF62 and ORF63 transcripts in rat DRG tissues after inoculation of wild-type VZV or VZV containing DHFR degrons. L4, L5, L6 DRG were isolated from VZV pOka (top), ORF4nDHFR (middle), or ORF63cDHFR (bottom) inoculated rats at 4-, 5-, and 7-dpi and used to prepare total RNA. RNA was then quantified and analyzed by TaqMan probes for expression of ORF62 (blue), ORF4 (red), ORF63 (green), and DHFR (yellow) transcripts compared to naïve uninoculated animals. RNA quantification was then normalized and analyzed by the 2^-ΔΔCt^ method relative to GAPDH. The dotted line (= 1) represents no change over GAPDH control. Data represents two similar experiments combined and averaged. Error bars: SD.
(TIF)
