# Supplementary material for: Varicella-zoster virus early infection but not complete replication is required for the induction of chronic hypersensitivity in rat models of postherpetic neuralgia
Source: PLoS Pathog. 2021 Jul 6;17(7):e1009689. doi: 10.1371/journal.ppat.1009689 (PMC8259975; doi:10.1371/journal.ppat.1009689)
Supplement: S1 Table — TaqMan primer/probe sets used in RT-qPCR analysis of VZV-infected rat DRG. 6-FAM (6-carboxyfluorescein). BHQ1 (black hole quencher 1). (DOCX) [file ppat.1009689.s001.docx]

**Supplementary Table 1. Primers for RT-qPCR.**

| # | Gene | Direction | Primer Sequence (5’ 🡪 3’) | Probe |  |  |  |
| --- | --- | --- | --- | --- | --- | --- | --- |
| 1 | ORF62 | Fwd | CCTTGGAAACCACATGATCGT | 6-FAM-TGCAACCCGGGCGTCCG-BHQ1 | | | |
|  |  | Rev | AGCAGAAGCCTCCTCGACAA |  |  |  |  |
| 2 | ORF4 | Fwd | CCTTCGGATGACTTTGCATT | 6-FAM-CTCCAGGCGAGGACTCCACA-BHQ1 | | | |
|  |  | Rev | TCGTTTGGAATACCGTGGAT |  |  |  |  |
| 3 | ORF63 | Fwd | GCTTACGCGCTACTTTAATGGAA | 6-FAM-TGTCCCATCGACCCCCTCGG-BHQ1 | | | |
|  |  | Rev | GCCTCAATGAACCCGTCTTC |  |  |  |  |
| 4 | DHFR | Fwd | CCATACCTGGGAATCAATCG | 6-FAM-ACGGACGATCGCGTAACGTG-BHQ1 | | | |
|  |  | Rev | CTTCATCCACCGACTTCACC |  |  |  |  |
