# Supplementary figures and images for: Varicella-zoster virus early infection but not complete replication is required for the induction of chronic hypersensitivity in rat models of postherpetic neuralgia
Source: PLoS Pathog. 2021 Jul 6;17(7):e1009689. doi: 10.1371/journal.ppat.1009689 (PMC8259975; doi:10.1371/journal.ppat.1009689)

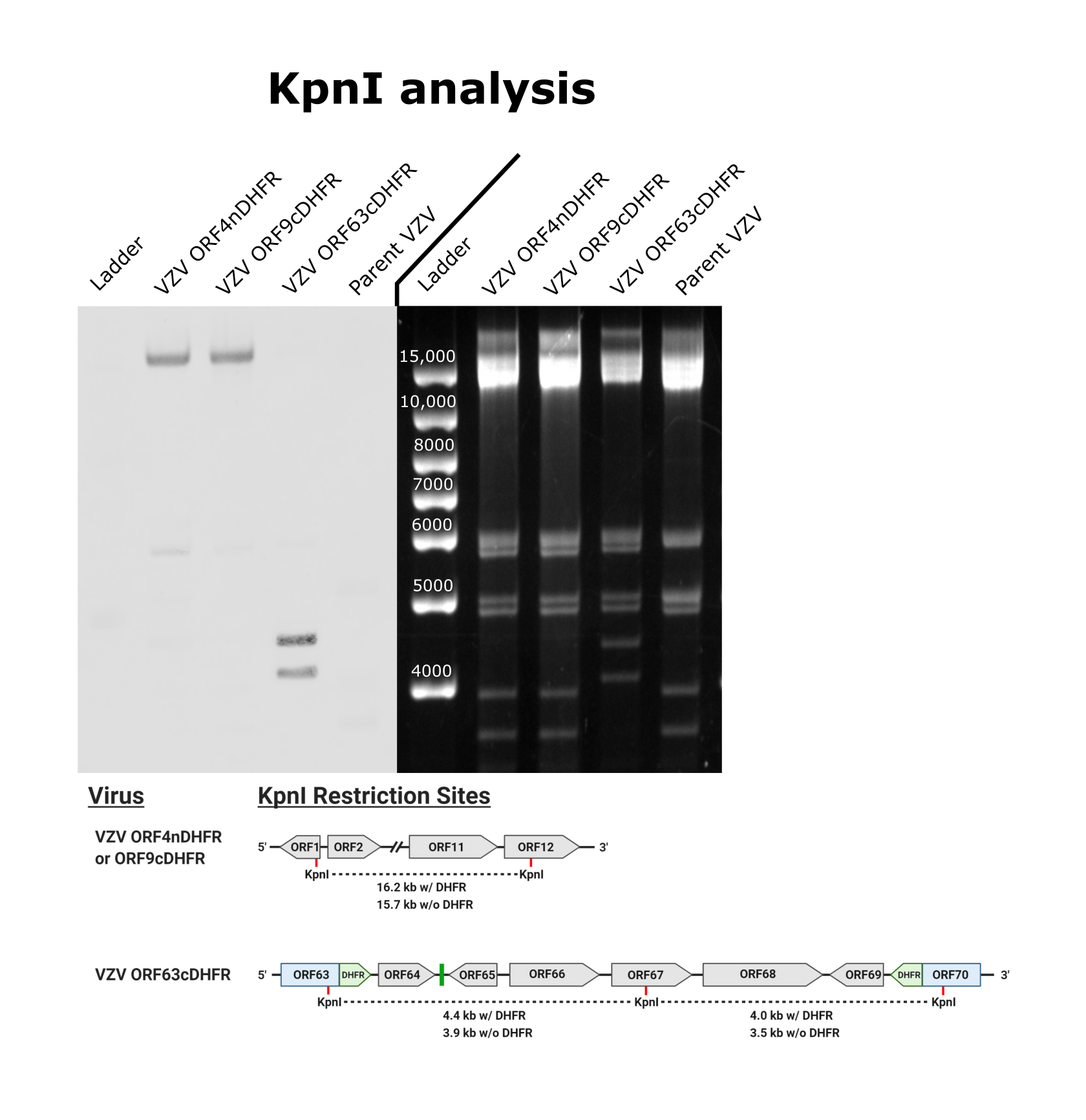

Supplement: S1 Fig — Southern blots (left) are aligned with the ethidium bromide-stained 1% agarose gel electrophoresis image of separated fragments following KpnI digestion of purified VZV nucleocapsid DNA from each DHFR degron inserted virus, or from virus derived from the parental VZV BAC (right). The DHFR probe generated by PCR predominantly hybridized to the same large DNA gel fragment for VZV ORF9cDHFR and ORF4nDHFR. For VZV ORF63cDHFR, two DNA fragments seen in other viruses (and not hybridizing the DHFR probe) increased in size by 480-bp and both hybridized the DHFR probe, with the larger representing ORF63 and smaller representing ORF70, respectively. A map of the regions of the DNAs for the expected KpnI DNA fragments in each virus is shown below the gel images with the expected fragment size indicated with and without the DHFR sequence insertion. The green vertical bar in the lower diagram for VZV ORF63cDHFR represents the position of the insertion of the BAC mini-F sequence (~8 kb) that self-excises with virus derivation and passage. A minor low abundance DNA fragment hybridizing the DHFR probe of ~6000-bp in size is present in every virus and was judged to be due to non-specific hybridization. Southern blot images were acquired on LICOR Odyssey in linear range. Created with BioRender.com. (TIF) [file ppat.1009689.s002.tif]

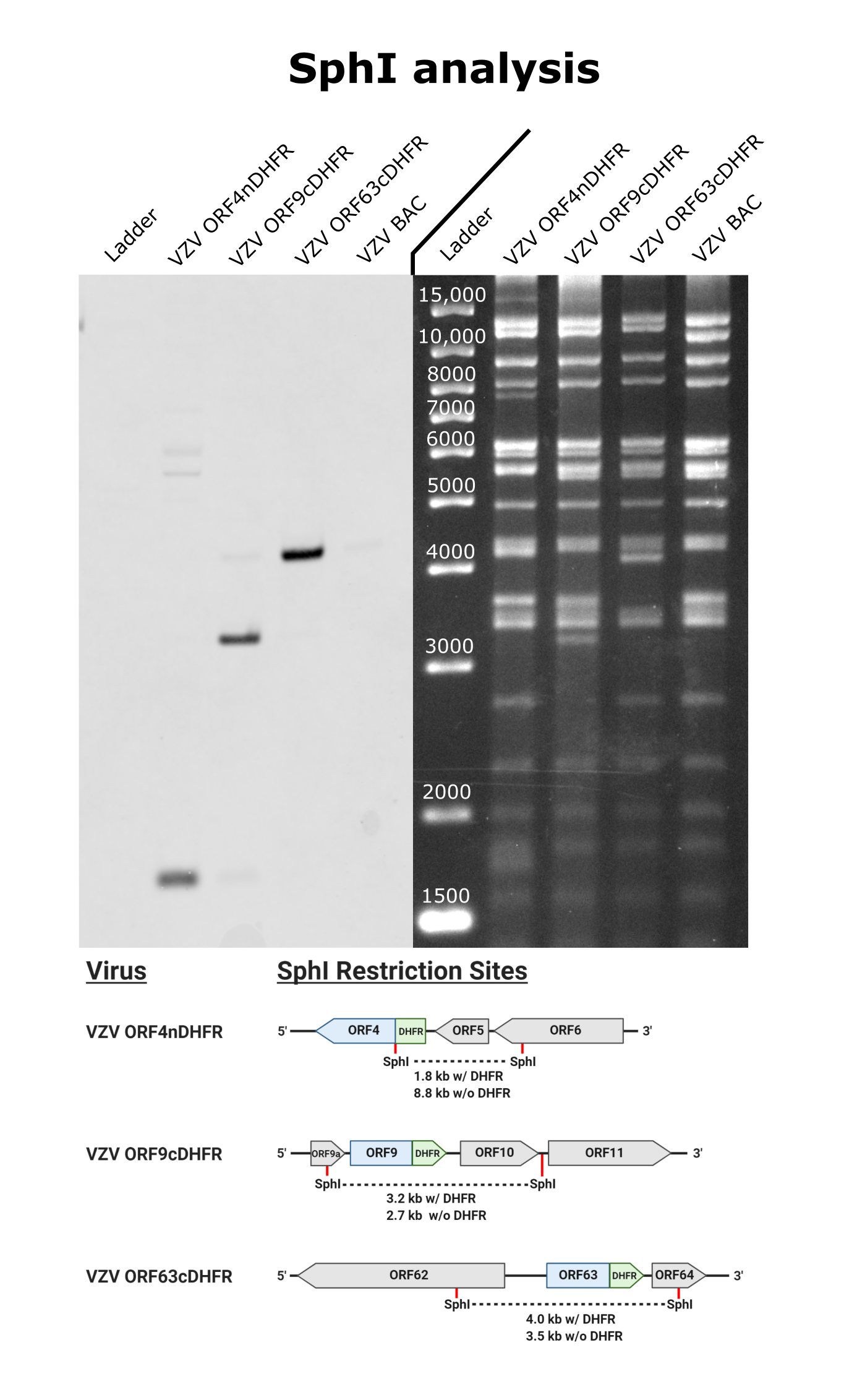

Supplement: S2 Fig — Southern blotting of 1% agarose DNA-separated SphI digested fragments with a DHFR-specific probe (left) and the ethidium bromide-stained VZV nucleocapsid DNA after gel electrophoresis (right). A map of the DNA fragments is shown at the bottom for each virus DNA as predicted from insertion at the correct sites for each virus. The map shows the predicted DNA fragment size with and without the degron sequence insertion. The sizes of a DNA ladder are shown in the composite image. The blots reveal that the degron insertions for each virus result in the increase of specific DNA fragment by 480-bp that then are the main fragments hybridizing the DHFR probe as predicted. Two ~6000 bp fragments hybridizing the DHFR probe at low levels for VZV ORF4nDHFR DNA are of sizes expected from partial digestion products at low levels in which the expected fragment is not restriction digested from the adjacent SphI DNA fragment. Created with BioRender.com. (TIF) [file ppat.1009689.s003.tif]

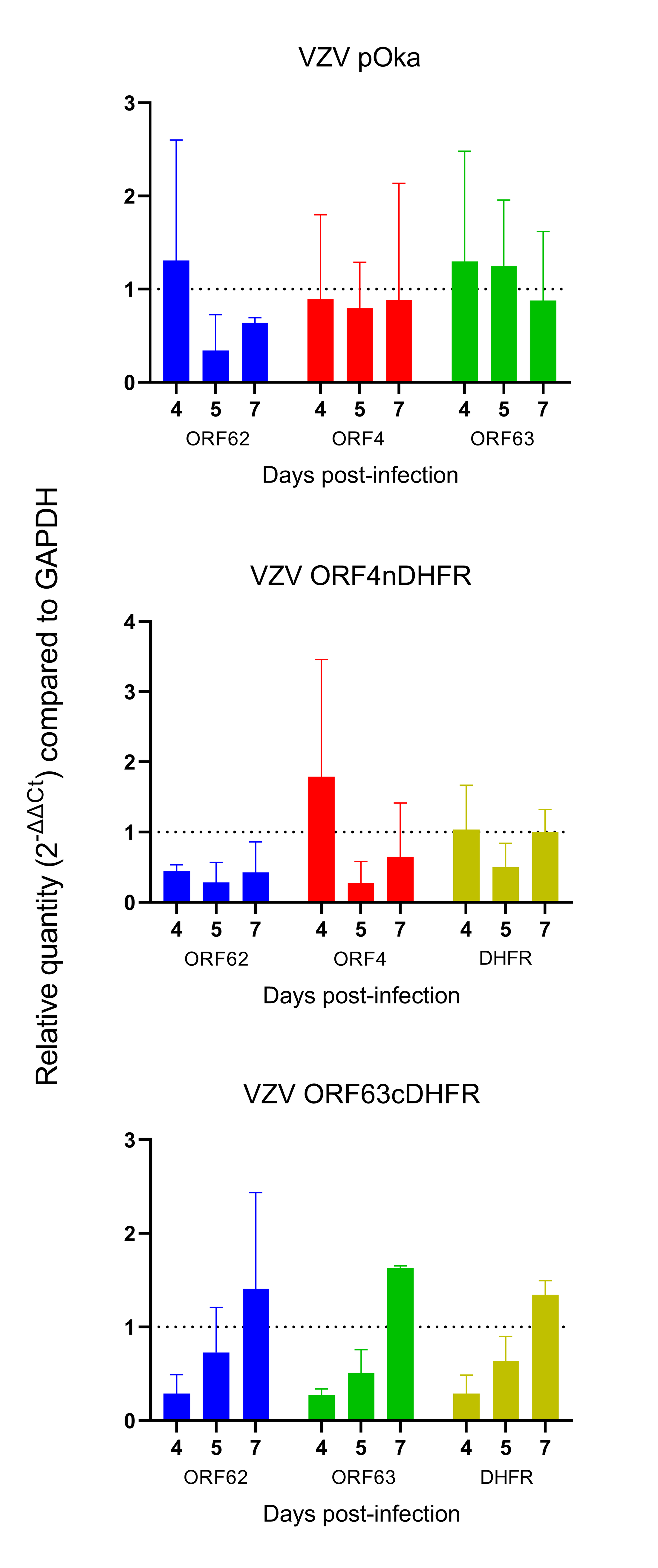

Supplement: S3 Fig — L4, L5, L6 DRG were isolated from VZV pOka (top), ORF4nDHFR (middle), or ORF63cDHFR (bottom) inoculated rats at 4-, 5-, and 7-dpi and used to prepare total RNA. RNA was then quantified and analyzed by TaqMan probes for expression of ORF62 (blue), ORF4 (red), ORF63 (green), and DHFR (yellow) transcripts compared to naïve uninoculated animals. RNA quantification was then normalized and analyzed by the 2-ΔΔCt method relative to GAPDH. The dotted line (= 1) represents no change over GAPDH control. Data represents two similar experiments combined and averaged. Error bars: SD. (TIF) [file ppat.1009689.s004.tif]
